# A data-driven approach to modeling cancer cell mechanics during microcirculatory transport

**DOI:** 10.1038/s41598-021-94445-5

**Published:** 2021-07-27

**Authors:** Peter Balogh, John Gounley, Sayan Roychowdhury, Amanda Randles

**Affiliations:** 1 Department of Biomedical Engineering, Duke University, Durham, NC USA; 2 Computational Sciences and Engineering, Oak Ridge National Laboratory, Oak Ridge, TN USA

**Keywords:** Computational models, Cancer

## Abstract

In order to understand the effect of cellular level features on the transport of circulating cancer cells in the microcirculation, there has been an increasing reliance on high-resolution in silico models. Accurate simulation of cancer cells flowing with blood cells requires resolving cellular-scale interactions in 3D, which is a significant computational undertaking warranting a cancer cell model that is both computationally efficient yet sufficiently complex to capture relevant behavior. Given that the characteristics of metastatic spread are known to depend on cancer type, it is crucial to account for mechanistic behavior representative of a specific cancer’s cells. To address this gap, in the present work we develop and validate a means by which an efficient and popular membrane model-based approach can be used to simulate deformable cancer cells and reproduce experimental data from specific cell lines. Here, cells are modeled using the immersed boundary method (IBM) within a lattice Boltzmann method (LBM) fluid solver, and the finite element method (FEM) is used to model cell membrane resistance to deformation. Through detailed comparisons with experiments, we (i) validate this model to represent cancer cells undergoing large deformation, (ii) outline a systematic approach to parameterize different cell lines to optimally fit experimental data over a range of deformations, and (iii) provide new insight into nucleated vs. non-nucleated cell models and their ability to match experiments. While many works have used the membrane-model based method employed here to model generic cancer cells, no quantitative comparisons with experiments exist in the literature for specific cell lines undergoing large deformation. Here, we describe a phenomenological, data-driven approach that can not only yield good agreement for large deformations, but explicitly detail how it can be used to represent different cancer cell lines. This model is readily incorporated into cell-resolved hemodynamic transport simulations, and thus offers significant potential to complement experiments towards providing new insights into various aspects of cancer progression.

## Introduction

While it is widely accepted that the hemodynamic transport of cancer cells in the microcirculation plays a key role in the etiology of cancer, the effect of cell structure and biomechanics on cell trajectory and the locations of spread is not well understood. Our knowledge is only cursory of how differences in cell parameters, which change based on cancer type, manifest at scale and in-turn influence preference for metastatic attachment location in the vascular tree. This is especially important given that characteristics of spread are known to depend on cancer type^1–4^. In silico approaches offer a compelling technique to investigate the complex interplay between cellular mechanics and transport due to their ability to provide precise control over cell parameters, microvascular topology and the complex fluid flow dynamics within. To-date, such approaches have mostly been utilized to model generic cancer cells, yet the necessary model components to resolve transport behavior that can distinguish between specific cell lines are largely unknown. Such a model opens the door to tackling long-standing questions^5^ and, importantly, on a cancer *type-specific* basis, including how new insights into the relationship between biomechanics, transport behavior, and disease progression can be exploited to guide new treatment approaches and diagnostic tools^5^, and how differences in cell deformability affect both margination behavior^6^ and adhesion dynamics^7,8^ towards augmenting predictability of attachment sites.

In order to build a cell-specific model that is robust and adaptable to represent different cancer types over a range of conditions, we need to establish the components of the cell that must be included in the model and how to tune their settings to match specific cancer types. In terms of cell mechanics, deformability is one critical characteristic of cancer cells affecting microcirculatory transport behavior^6–10^, and this attribute specifically is known to vary based on cancer type^11,12^. To-date, researchers have established many methods with varying levels of complexity to accurately model features like cell deformability^5,13,14^. Popular among these methods include membrane model-based approaches in which a membrane encloses a viscous fluid, and generally consist of either single-membrane or nucleated cell models. The latter are also referred to as compound cells, with the outer membrane and nucleus modeled as two separate membranes. With such approaches the deformability is controlled via the membrane stiffness(es), and the precedent of using these models in a more generic manner to complement experimental observations has been established over the last few decades. The liquid drop approach with membrane surface tension has been used to model both single membranes^15–19^ and compound cells^20–23^, many of which have been used to interpret mechanical properties of cells from experimental data. More detailed membrane models validated against experiments have been developed which also incorporate elasticity and other complexities as well. Rejniak^24^ developed a 2D compound cancer cell model with elastic membranes, which included the capability of modeling cell-cell adhesion and cell proliferation. Leong et al.^25^ used a similar 2D approach to investigate breast cancer cells entering a constricted microchannel, including both surface tension and elasticity. Au et al.^26^ used a 3D single-membrane model to simulate cancer cells with membranes modeled by non-linear elastic bead-spring networks to aid in their experimental study of cancer cells and clusters squeezing through capillaries. Barber and Zhu^27^ used a 2D single-membrane model with interconnected Kelvin-Voight elements, and observed good agreement with experiments involving breast cancer cells deforming through a microfluidic channel.

Approaches have also augmented membrane models with components to model internal cytoskeletal networks, and with these the deformability is also controlled by network components such as filament stiffnesses. Ujihara et al.^28^ developed a model using elastic springs to represent the cytoskeleton which connected compound cell membranes each modeled by 2D elastic meshes, and were able to reproduce experimental results from tensile tests of fibroblasts. Ghaffari et al.^29^ used a method with elastic cytoskeleton components including actin filaments, intermediate filaments, microtubules, and actin binding proteins, and were able to reproduce experimentally observed behavior as well^30^. Lykov et al.^31^ developed a compound cell model including viscoelasticity as well as an internal cytoskeleton effectively grown based on principles underlying its formation, and was validated for breast epithelial cells against microfluidic experimental data.

Evidently, with each of these works the different cell models employed have been shown to reproduce behavior of specific cells under set circumstances. However, what is lacking is an understanding of what level of complexity is really required for the model to accurately account for microcirculatory transport behavior. That is, what are the features necessary in a cell model to capture behavior measured experimentally, and moreover, how do we modulate these parameters so that the model can be tuned to different cancer types.

In terms of practical application, studying cell mechanics and influence on macroscopic behavior like transport requires not just any tuneable cell model, but one that can be utilized in a computationally tractable way to account for effects of cellular interactions and complex dynamics. A major strength of membrane-model based approaches is their computational efficiency, which is an important consideration for large-scale simulations which resolve cell interactions and fluid flow in complex 3D vasculatures. Over the past decade, such approaches have been integrated into in silico frameworks for 3D microcirculatory flow modeling, which has been a rich area of research^32–40^. Among the different methods developed, the immersed boundary method (IBM)^41,42^ has been a popular choice owing to its computational efficiency in accurately modeling complex 3D flows. With the IBM, separate solvers for the fluid mechanics and solid mechanics are two-way coupled. Numerical methods such as the lattice Boltzmann method (LBM) or finite volume method (FVM) are commonly used to solve the governing flow equations, and methods such as the finite element method (FEM) are used to determine the stresses generated in cell membranes as they deform in response to the fluid flow. For modeling deformable cells the Skalak constitutive law^43^ is a popular choice for the stress–strain relationship^33,36,38^. While this law was originally designed for red blood cells (RBCs), works have also used it to represent generic cancer cells by simply considering them to be spherical when undeformed, larger, and more stiff^44–47^. This idea has also been used with other membrane models and *in silico* approaches as well^32,34,39^. Use of the Skalak model is not only convenient within an IBM-based framework, but there is some physical basis for modeling cancer cells in that it is a strain-hardening model^48^, and filamentous actin which is a main contributor to deformation resistance^5^ is known to exhibit strain-hardening behavior^49,50^.

While such modeling efforts involving generic cell models have provided broad insights into cell transport behavior^44,46,47,51^, there has been no established procedure for tuning such computational models to a specific cancer cell type. Moreover, there is a lack of systematic studies comparing cell models with different components included to optimally fit experimental data over a range of conditions. Resolving these open questions is important towards establishing the accuracy of a modeling approach that can distinguish between cancer cell types while providing an efficiency that allows fast tuning to different cell lines and assessing behavior on a macroscopic level. Furthermore, an approach which marries experiments and simulations in a way that enables efficient model tuning can provide a mutually beneficial platform for both aiding in simulation efforts as well as designing in vitro experiments. Numerous devices have been designed for determining cell properties which examine behavior under both flowing conditions (e.g.^11,52,53^) as well static/quasi-static conditions (e.g.^12,54,55^). Given the complex structure of cancer cells and the potential for rate-based property dependence, establishing the accuracy of a model through comparing with behavior from both of these perspectives is important to properly identify model components needed to capture observed behavior. Additionally, CTCs are exposed to a range of conditions during microcirculatory transport, and integrating data from different experiments with an in silico model can expand the range of application for both experimental properties determined as well as the modeling approach.

To overcome the current limitations of existing models, and towards addressing the critical issues described, in the present work we develop and validate an efficient, data-driven, generalizeable approach to simulate the microcirculatory flow transport of specific cancer cell lines. We consider both single membrane and nucleated cell models. As discussed, both of these are commonly used in the literature for modeling cancer cells, although circumstances warranting the use of one over the other remain unclear. First, we investigate the degree to which each can capture behavior of murine leukemia cells (L1210) deforming through a microfluidic constriction. We then verify our findings and expand the range of application by comparison with separate experimental data in the literature. We also describe a spring based cyto- and nucleo-skelelton model to augment the nucleated cell model, and compare accuracy. Lastly, we use the nucleated cell model to represent cancer cells from a different line, namely a murine lung cancer line. We outline a systematic approach to parameterize different cell lines to optimally fit experimental data over a range of deformations.

## Materials and methods

### Cell-resolved fluid flow simulations

Simulations are performed using HARVEY, a 3D lattice Boltzmann method (LBM)-based computational fluid dynamics solver^37,40,56–58^. HARVEY has been used to study a wide range of physiological applications, such as the hemodynamics of a growing cerebral aneurysm^59^, computing the ankle-brachial index for diagnosing conditions such as peripheral artery disease^60^, cell adhesion in microvessels and impacts on wall shear stress^47^, and large-scale RBC simulations in a cerebral vasculature^40^. For modeling flows of deformable cells, HARVEY utilizes the immersed boundary method (IBM), and with this approach cancer cells are modeled as membranes of infinitesimal thickness surrounded by and enclosing a viscous fluid with which they flow. The IBM facilitates a two-way coupling between cell deformation in response to the fluid flow, and the stress imparted by the deforming cell to the fluid. The foundation of the LBM fluid dynamics solver is the lattice Boltzmann equation, with this fluid-structure interaction (FSI) incorporated by means of a distributed body force^61^:

1 $$\begin{aligned} f_i\left( \mathbf{x} +{{\varvec{c}}}_i \delta t, t+\delta t\right) = f_i\left( \mathbf{x} ,t\right) - \frac{1}{\tau }\left( f_i\left( \mathbf{x} ,t\right) -f_i^{eq}\left( \mathbf{x} ,t\right) \right) - h_i\left( \mathbf{x} ,t\right) \delta t \end{aligned}$$

Here, $f_i$ is the particle distribution function, $f_i^{eq}$ is an approximation of the Maxwell-Boltzmann equilibrium distribution, $h_i$ is the distributed body force, and $\delta t$ is the time step.

The LBM is a mesoscopic approach to numerically solve the Navier–Stokes equations^62^, where the fluid is represented by this particle distribution function that evolves following Eq. (1). We solve Eq. (1) on a uniform Eulerian lattice using a D3Q19 velocity discretization, and ${{\varvec{c}}}_i$ is the velocity of the *i*th population in accordance with this scheme^61^. The macroscopic fluid density is the 0th order moment:

2 $$\begin{aligned} \rho = \sum _i f_i\left( \mathbf{x} ,t\right) \end{aligned}$$

and the momentum is the first order moment, from which we get an expression for the macroscopic velocity in the presence of a body force:

3 $$\begin{aligned} {{\varvec{v}}} = \frac{1}{\rho } \sum _i {{\varvec{c}}}_i f_i\left( \mathbf{x} ,t\right) + \frac{\delta t}{2\rho } {{\varvec{g}}} \end{aligned}$$

where ***g*** is a body force in the form of force per volume. With the simulation technique employed here, this body force term is used to distribute the stress generated in the cell membranes as they deform to the fluid points on the Eulerian lattice. The relationship between this body force and the force distribution associated with the *i*th population used in Eq. (1) is determined following^63^:

4 $$\begin{aligned} h_i = \left( 1-\frac{1}{2\tau }\right) w_i \left[ \frac{{{\varvec{c}}}_i-{{\varvec{v}}}}{c_s^2} + \frac{{{\varvec{c}}}_i \cdot {{\varvec{v}}}}{c_s^4} {{\varvec{c}}}_i\right] \cdot {{\varvec{g}}} \end{aligned}$$

where $w_i$ denote the standard D3Q19 weights of the *i*th population. More complete details on the LBM implementation are provided in the Supplementary Information.

#### Cancer cell membrane model

**Figure 1:**
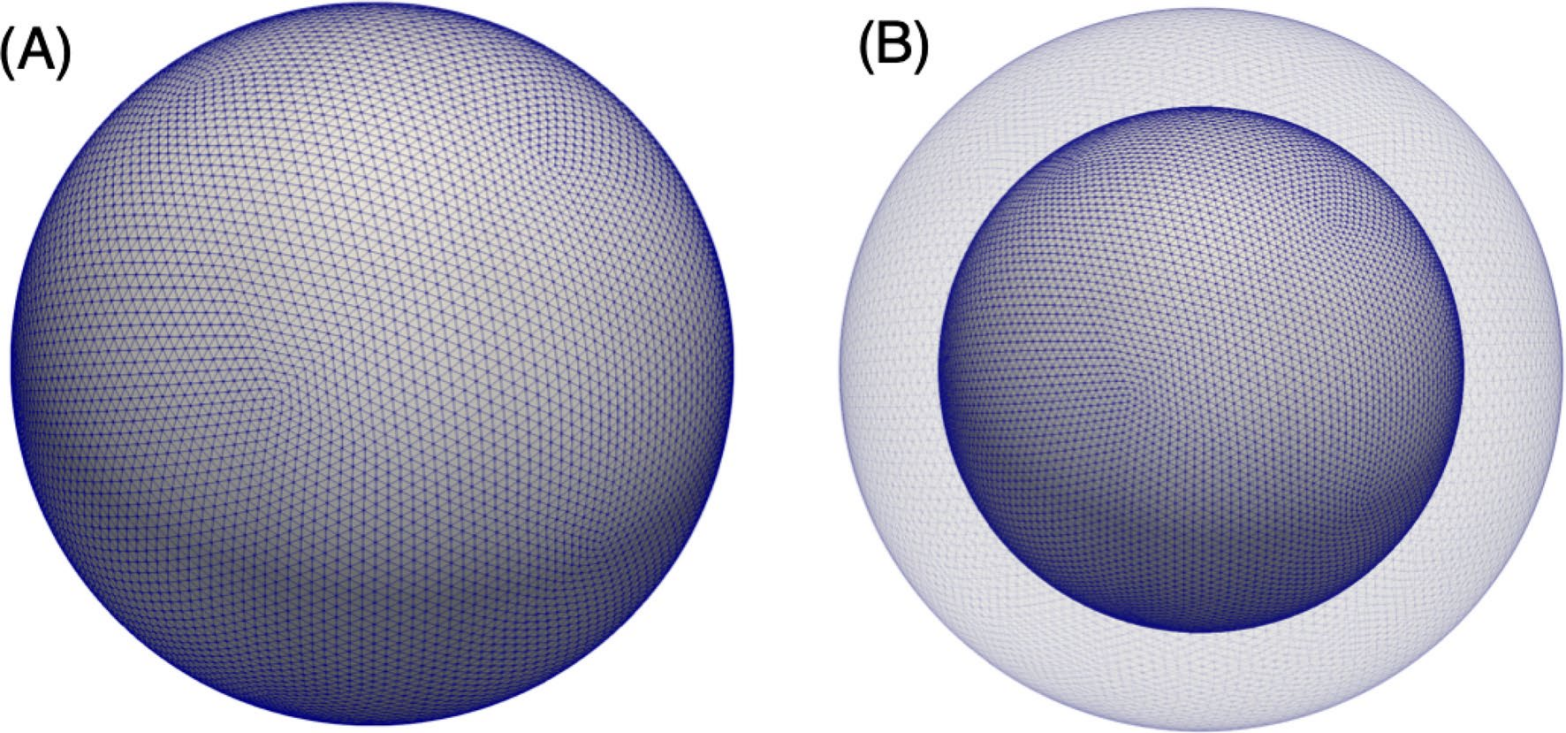
Lagrangian mesh of triangular elements as applied to the **(A)** single-membrane and **(B)** nucleated cell models. Here, each cell membrane is discretized by 20,480 triangles.

Cancer cell membranes are defined by a Lagrangian mesh of triangular elements, representative examples of which are given in Fig. 1 for the single membrane and nucleated cell models. To determine the cell membrane deformation between timesteps, the Lagrangian membrane velocity (***V***) is first interpolated from the Eulerian fluid velocity (***v***) using the three-dimensional Dirac delta function:5for Lagrangian membrane vertex location ***X*** and Eulerian fluid lattice location ***x***, and the integral is over the fluid volume. We approximate $\delta$ using a cosine function which spans four Eulerian lattice points around the cell membrane^41^:

6 $$\begin{aligned} \delta \left( {{\varvec{x}}}-{{\varvec{X}}}\right) = \frac{1}{64\delta x^3} \prod _{i=1}^3 \left[ 1+\cos \frac{\pi }{2\delta x}\left( x_i - X_i\right) \right] \end{aligned}$$

where $\delta x$ gives the spacing on the Eulerian fluid grid.

The resulting force in the membrane due to the deformation is determined using the finite element method (FEM). With this, we model the membrane as resisting shear and area dilation following the strain energy function of Skalak et al.^43,64^:

7 $$\begin{aligned} W_s = \frac{G_s}{4}\left[ \left( I_1^2+2I_1-2I_2\right) +CI_2^2 \right] \end{aligned}$$

where $G_s$ is the membrane shear elastic modulus, and $I_1$ and $I_2$ are the strain invariants of the Green strain tensor. *C* is a constant directly related to the area dilation modulus, and a large value of *C* results in small area dilation^64^. This is a commonly used model for red blood cells (RBCs) with this type of numerical approach^33,35,36,38,58^, and in the present work we adopt it to model cancer cells. With this approach we vary the shear elastic modulus ($G_s$) to modulate the cell stiffness. We also model the membrane as possessing resistance to bending, the energy of which is modeled following Helfrich^65^:

8 $$\begin{aligned} W_b = \frac{E_b}{2}\int _S \left( 2\kappa -c_0 \right) ^2 dS \end{aligned}$$

where $E_b$ is the bending modulus, $\kappa$ is the mean curvature, $c_0$ is the spontaneous curvature, and *S* is the surface area. In the present work since we are modeling cells generally regarded as being stiffer than RBCs; consistent with previous work^29^, we use $E_b=1\times 10^{-18}\,\,\hbox {J}$.

Loop elements are used as a subdivision surface^66–69^ for the FEM membrane force calculations, which determines elemental strains based on 12 surrounding triangular surface elements. This provides improved stability for cases in which large and highly localized surface forces can occur, such as that which may be experienced by membranes undergoing large and complex deformations. The FEM calculations ultimately result in forces at each Lagranian vertex, ***G***, which are then spread back to the Eulerian lattice using the same delta function in Eq. (6):

9 $$\begin{aligned} {{\varvec{g}}}= \int _S {{\varvec{G}}} \delta \left( {{\varvec{x}}}-{{\varvec{X}}}\right) d{{\varvec{X}}} \end{aligned}$$

Additional details on the numerical method and the deformable cell model used with the present work are provided in the Supporting information. They are also described in our previous works, which include other validations^37,57,58^.

The modeling approach employed here essentially considers an incompressible cytoplasmic fluid, which is a common assumption in the literature with simulation-based analyses (e.g.^44,46,47,51^). The physical justification for this is rooted in experiments which have demonstrated this characteristic under many common physiological circumstances^70,71^, and is primarily due to the lipid bilayer which comprises the plasma membrane and encloses the cytoplasm, and that this behaves as an incompressible fluid^72^. With many physiological processes, however, fluid exchange across cell membranes is known to occur when triggered by relevant factors. Such processes can include cell migration through vessel walls and into tissue, and also for drug uptake towards combating cancer, among others. Such trans-membrane fluid exchanges typically alter the overall cell volume, though these are relatively passive processes compared to the timescales relevant to the present work. That is, here our passage times are on the order of milliseconds, while the timescales associated with processes such as cell migration are typically orders of magnitude larger^73–75^. Such timescale discrepancies can be even more pronounced with trans-membrane particulate transport, as works have shown that cancerous cells as compared to healthy cells can have further decreased permeability which presents as a barrier to drug delivery^76–79^. The target applications for the models developed in the present work are flow and transport simulations of cells through the microcirculation, and thus those in which membrane permeability does not play a role. For circumstances in which this does play a role, modifications to the models would be warranted.

#### Using an adaptive spring-based approach to model the cyto- and nucleo-skeleton components

We augment the nucleated cell model with a network of elastic springs meant to mimic the mechanical structure of the cyto- and nucleo-skeleton. We note that this model is simplistic in that it does not directly capture the more complex structures which comprise the actual interior of a cancer cell. However, this provides an additional measure beyond the membrane model alone to directly capture the resistance to deformation caused by internal skeletal networks themselves.

**Figure 2:**
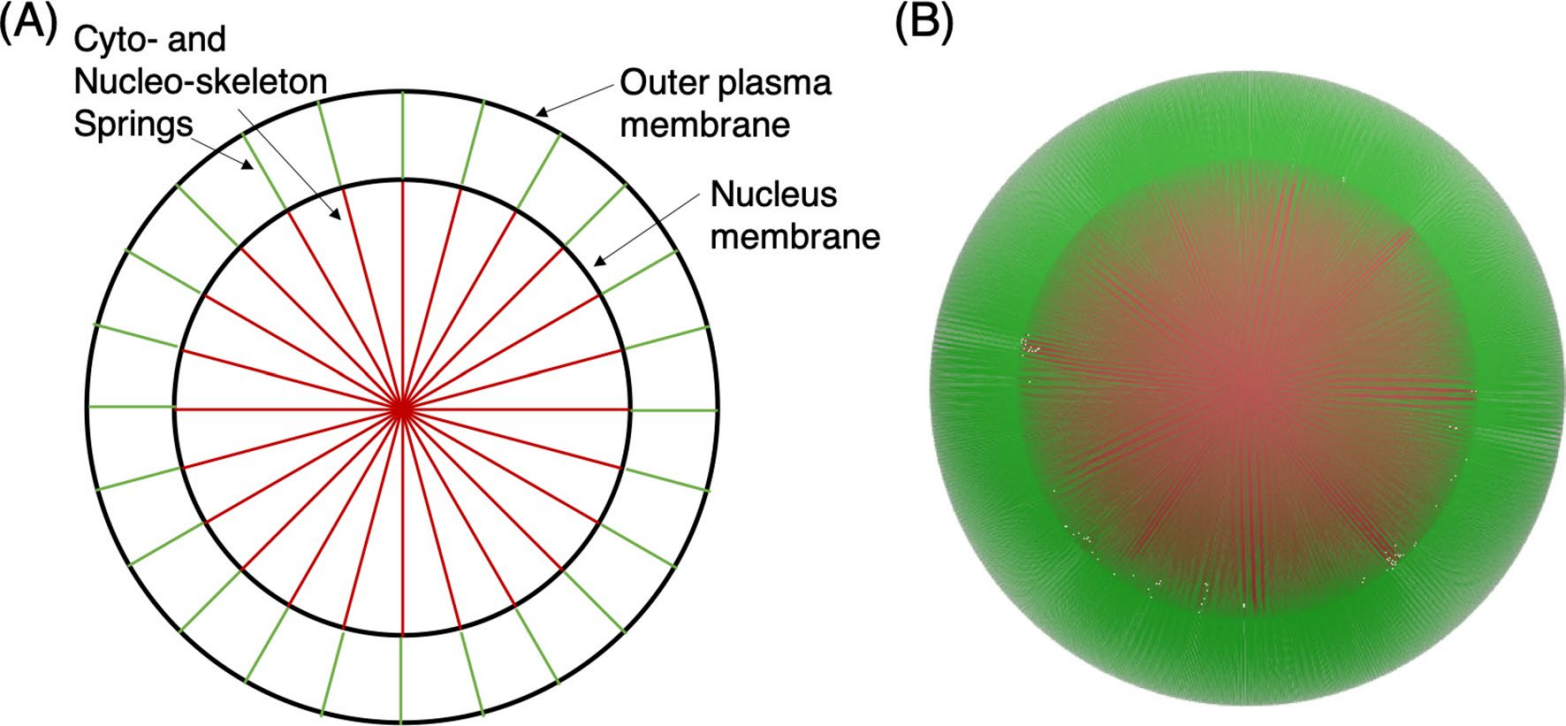
Components of the nucleated cell with cyto- and nucleo-skeleton model. **(A)** Schematic depiction of each component, including the outer plasma and nucleus membranes, as well as the cyto- and nucleo-skeleton springs. Green springs connect from the outer membrane to the nucleus, and red springs connect opposing locations on the nucleus membrane. **(B)** 3D cell model depicting the spring configuration in the undeformed state, with 20,480 springs for each region.

Our model includes springs that connect from the outer plasma membrane to the nucleus, as well as inside the nucleus between opposing locations. Springs are connected to each element of the Lagrangian mesh for the cell membranes. Figure 2A gives a schematic depiction of this structure for a cancer cell in its undeformed state, and Fig. 2B shows this for a simulated cell. The force in each spring is determined from Hooke’s law as $F=kdL$, where the spring constant $k = EA/L_0$, and $dL = L(t) -L_0$ which gives the spring deformation relative to the initial reference length $L_0$. Additionally *E* is the Young’s modulus and *A* is the elemental area associated with the spring. Hookean springs have been used in other works (e.g.^28^), which have demonstrated the ability to reproduce behavior of actin filaments in this context.

As the cell deforms to enter the constricted microchannel the temporary concavity of the membranes can result unphysical behavior where the springs connect membrane locations by going outside of the cell. To overcome this, a technique is developed where springs adapt to the instantaneous cell structure. The basic premise as applied to each surface element at which one end of the spring is connected is to project in a direction normal to the local surface and connect the other end to the element intersected. As such, springs connecting from the outer membrane can connect either to elements on the nucleus or other elements on the outer membrane, based on the instantaneous membrane configurations and curvatures. This adaptive technique is discussed in more detail in “Adaptive cyto- and nucleo-skeleton components capture large deformation behavior with same accuracy as nucleated cell model”, and additional details on this model as well as the algorithm to implement it are provided in the Supplementary Information.

### Integrating simulations with experiments to model specific cell lines

During microcirculatory flow transport, cancer cells experience a range of conditions, from flowing in larger microvessels where they interact with red blood cells (RBCs), to small microvessels where they have to squeeze to flow through. In developing a cancer cell model which can capture this range of dynamic flow behaviors, focusing on the more extreme deformation and transport provides a robust model validation to accurately capture cell transport behavior over the range of conditions experienced during microcirculatory flow transport.

**Figure 3:**
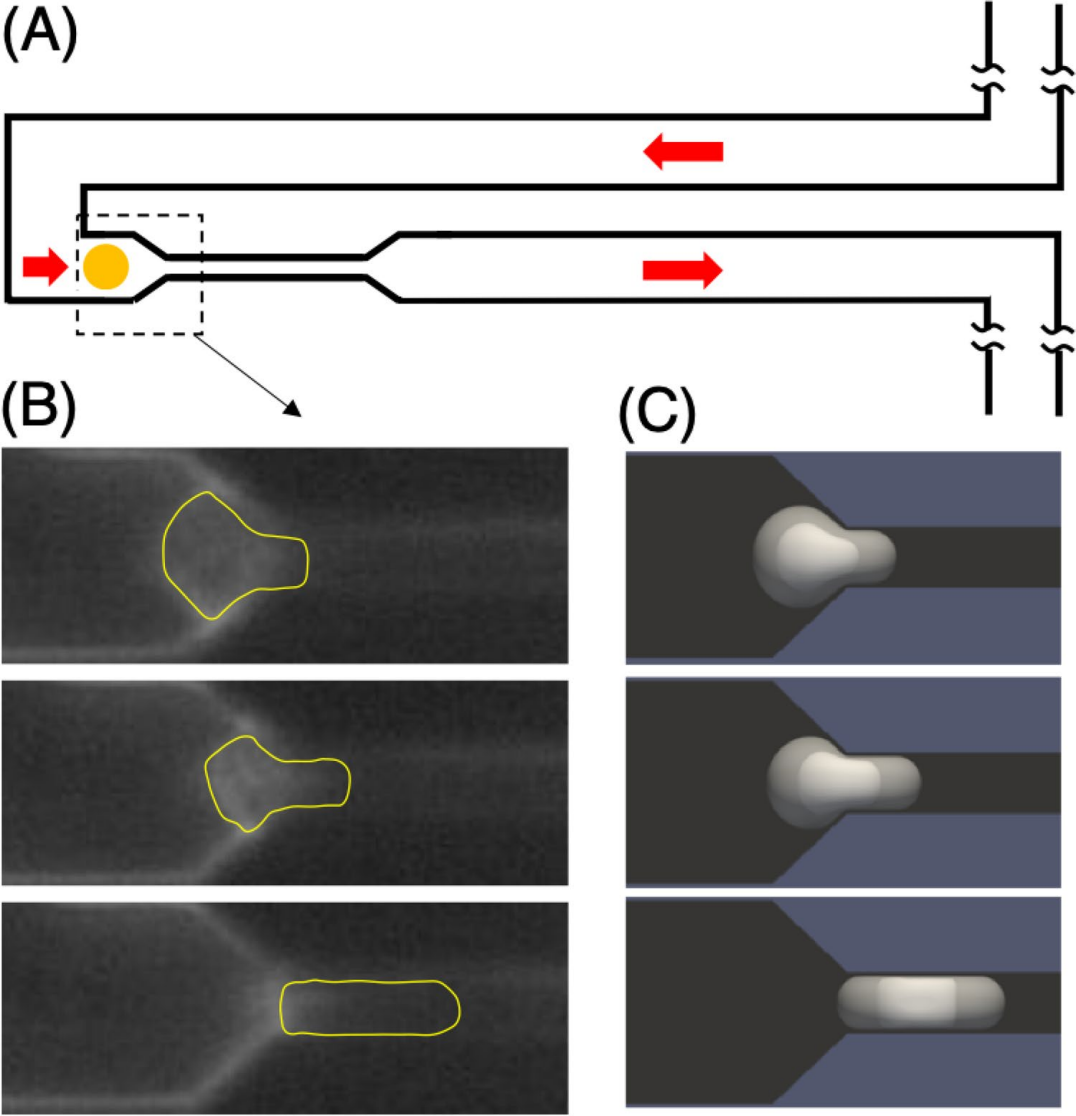
Depiction of the experiments performed in^11^ and the simulations. **(A)** Schematic depicting the experimental setup where cancer cells were driven through a microfluidic constriction. **(B)** Images from a video provided to us by the authors of^11^ showing an L1210 cell squeezing to enter the constricted microchannel, in sequence. The yellow outline is approximate, and was added to help illustrate the shape and position of the cell as it entered the constriction. **(C)** Images from our simulations of a nucleated cell squeezing to enter the microfluidic constriction.

To this end, simulations are performed to recreate the experiments of Byun et al.^11^, where individual cancer cells were driven through a microfluidic constriction device by an applied pressure difference, and passage times were reported. A schematic is provided in Fig. 3 depicting the experimental setup, along with images from a video of an experiment provided to us by the authors of Byun et al.^11^. These show a murine lymphoblastic leukemia cell (L1210) squeezing to enter the constricted microchannel, and to help illustrate the shape and position of the cell an approximate outline was added in yellow. Also provided are snapshots from a simulated cell using the nucleated cell model, exhibiting qualitatively similar behavior. As discussed later in “Nucleated cell model accurately captures cell-specific transport behavior by modulating the nucleus stiffness ratio” , the cell passage time through the device is dominated by this entry process illustrated here, in which the cell undergoes time-dependent large-deformation. Accurately resolving this from a modeling perspective is highly non-trivial, yet critical to capturing the transport behavior and passage time of cells through the device.

In the experiments of^11^, cancer cells were fed into the portion of the device depicted in Fig. 3A from a reservoir region of controlled pressure. The exit region of this schematic was connected to another region fed into a low-pressure reservoir. In the present work we consider cells from the L1210 line as well as those from a metastatic murine lung cancer cell line. For these cells lines, in Byun et al.^11^ the difference between pressures in the reservoirs was 0.9psi, with a total flow rate through the device of $25 \upmu \hbox {L}/\hbox {h}$. For L1210 cells modeled with a nucleus and outer plasma membrane, the diameter of each membrane is such that the ratio of nuclear volume to cell volume (karyoplasmic ratio) is approximately 38%, as has been reported in the literature for such cells^12^.

The schematic in Fig. 3A depicts the portion of the device that we have modeled. The constriction region is $50\,\,\upmu \hbox {m}$ in length, with cross-section dimensions of $6\,\upmu \hbox {m} \times 15\,\upmu \hbox {m}$. Upstream and downstream of this constriction the cross-section dimensions of the main channel are $20\,\upmu \hbox {m} \times 15\,\upmu \hbox {m}$, with a total length we approximate as $1000\,\upmu \hbox {m}$. In our model we assume that the pressure is constant at the inlet and outlet to the main channel. The cell is observed to rapidly flow through the main channel, and then significantly slows down as it squeezes to enter the constriction region. During this entry period, the pressure immediately upstream of the cell increases due to the temporary increase in resistance and decrease in flow rate. After entry has completed the cell quickly passes through the constriction region.

In order to adequately resolve both the fluid flow field and the cell membrane as it deforms through the constriction, we use a lattice spacing of $0.15\,\upmu \hbox {m}$ for the LBM fluid solver and a Langrangian mesh comprised of 20,480 elements for each cell membrane. This was found to be sufficient to properly capture the dynamics of the deforming cancer cell in our preliminary resolution testing as detailed in the Supplementary Information, and further validated by the experimental comparisons presented later. Additionally, we model a fluid with density $1060\,\,\hbox {kg}/\hbox {m}^3$ and kinematic viscosity $7.8\times 10^{-7}\, \hbox {m}^2/\hbox {s}$^11^. With the IBM-based approach employed in this work, this viscosity is for the background fluid which conveys the cell. Any contribution to the viscosity from the cell as it deforms will manifest in terms of a body force that gets generated through the FEM and added to the fluid via the IBM [i.e. $\mathbf{g}$ in Eqs. (4) and (9)].

Quantitative comparisons with Byun et al.^11^ involve the passage time of the cell only through the constriction region. As such, the portion of the simulation we utilize consists of the cell squeezing to enter and then transiting through the constriction region. Owing to the significant computational cost in performing a fully resolved 3D simulation of the entire geometry in Fig. 3A, we incorporate a multi-resolution modeling approach whereby the full 3D model is only utilized in the vicinity of the constriction region. This 3D model is then coupled to a lumped parameter model for the upstream and downstream regions. The premise behind this approach is to provide an efficient means of mimicking the dynamic change in pressure difference over the cell during the entry process to the constriction due to the temporary resistance increase previously mentioned.

With our multi-resolution approach, the 3D model consists of a constriction region that is $25\,\,\upmu \hbox {m}$ in length, and inlet and outlet regions totaling $50\,\,\upmu \hbox {m}$ in length. The remaining channel lengths, namely $950\,\,\upmu \hbox {m}$ for the main channel and $25\,\,\upmu \hbox {m}$ for the constriction region, are modeled with the lumped parameter model. For this lumped model we consider Boussinesq’s solution^80^ for pressure driven flow through a rectangular channel with cross-section *h* x *l*, where the pressure gradient *G* is related to the flow rate *Q* by:

10 $$\begin{aligned} G=Q\left[ \frac{h^3l}{12\mu } - \frac{16h^4}{\pi ^5\mu } \sum _{n=1}^\infty \frac{1}{\left( 2n-1\right) ^5}\frac{\cosh \left( \beta _nl\right) -1}{\sinh \left( \beta _nl\right) } \right] ^{-1} \end{aligned}$$

where $\mu$ is the dynamic fluid viscosity, and $\beta _n=(2n-1)\pi /h$ . Using the flow rate determined from the 3D model at each time step, we calculate *G* from Eq. (10) and use this with the appropriate channel lengths and dimensions to determine the pressure to be applied at the inlet to the 3D model at the next time step. We utilize a proportional-integral (PI) controller^81^ to alleviate instabilities in coupling the 3D and lumped parameter models. The full details of the approach, including validation with the full model, are provided in the Supplementary Information.

In terms of using this experimental data to model specific cell lines, ideally it would be best to compare against the full 3D features of the cell observed experimentally in addition to the passage time. While we have validated the 3D time-dependent membrane shapes for the nucleated cell model in a previous work^37^ against an alternate model implementation^44^, such data was not available for the experiments we are recreating here. Thus, to add a measure of robustness with regard to validation and our findings in recreating the experiments of Byun et al.^11^, in the “Recreating a separate experiment to verify cell properties and interrogate model accuracy under different conditions” we describe an additional, separate experiment we have recreated to further interrogate our model. We also note here that while there are other modeling paradigms which exist, what we have employed in the present work is common in the literature among simulation-based works^32–36,38,39,44^ as noted in the Introduction. As such, the findings and novel contributions of this work presented and discussed in the subsequent sections enable other researchers to model cancer cells undergoing large deformation during microcirulatory flow transport, and importantly with characteristics to distinguish between different types of cancer.

#### Recreating a separate experiment to verify cell properties and interrogate model accuracy under different conditions

We also perform simulations that recreate the experiments performed in Guo et al.^54^ involving L1210 cells. The purpose of comparing with an additional, separate experiment is this provides a verification of our approach to determine the cell properties for the model using experimental data from Byun et al.^11^. Moreover, as discussed in the Introduction, this experiment provides an additional perspective from which to interrogate our model given the quasi-static conditions under which cell properties were determined, which complements the more dynamic process in Byun et al.^11^.

**Figure 4:**
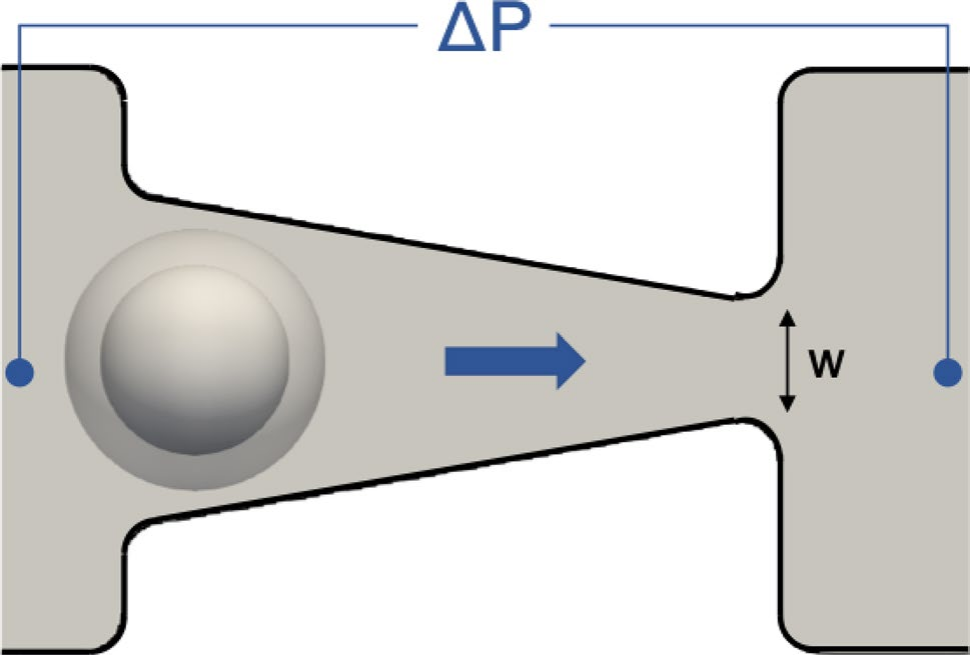
Snapshot from a simulation of a nucleated cell within the microfluidic ratchet device designed to recreate the experiments of Guo et al.^54^. L1210 cells were driven through device by an applied pressure difference $\Delta P$, as shown. The minimum $\Delta P$ required for the cell to pass through different pore sizes (*w*) was determined and compared to that reported in Guo et al.^54^.

The experiments performed in Guo et al.^54^ involved L1210 cells being driven by a controlled pressure difference through a microfluidic ratchet device designed by the authors of that work. Figure 4 provides a shapshot from one of our simulations of an L1210 cell within the device. In Guo et al.^54^, the minimum pressure difference across the cell required for it to pass through the narrow opening was determined, referred to as the threshold pressure ($P_{thresh}$), in a gradual quasi-static manner. We model the device with four different funnel pore sizes(*w*) of $6.9\,\,\upmu \hbox {m}$, $7.8\,\,\upmu \hbox {m}$, $8.8\,\,\upmu \hbox {m}$, and $9.8\,\,\upmu \hbox {m}$, and for each determine the $P_{thresh}$ value required for the cell to pass through. We consider cells with a diameter of $15.6\,\,\upmu \hbox {m}$ as reported in Guo et al.^54^, and compare the $P_{thresh}$ values determined from the simulations against the experimental data. We consider a nucleus with a diameter of $11.3\,\,\upmu \hbox {m}$, in line with the aforementioned karyoplasmic ratio reported for such cells in the literature.

## Results and discussion

### Single-membrane model captures transport behavior under limited circumstances

We first consider an L1210 cell modeled as a single plasma membrane (Fig. 1A). We modulate the cell stiffness by varying $G_s$ in the membrane constitutive law [Eq. (7)]. Generally speaking cancer cells are known to be much stiffer than RBCs, and healthy RBCs are generally on the order $5\,\,\upmu \hbox {N/m}$^43^. We use this as a reference point from which to choose values for modeling the less deformable cancer cells.

**Figure 5:**
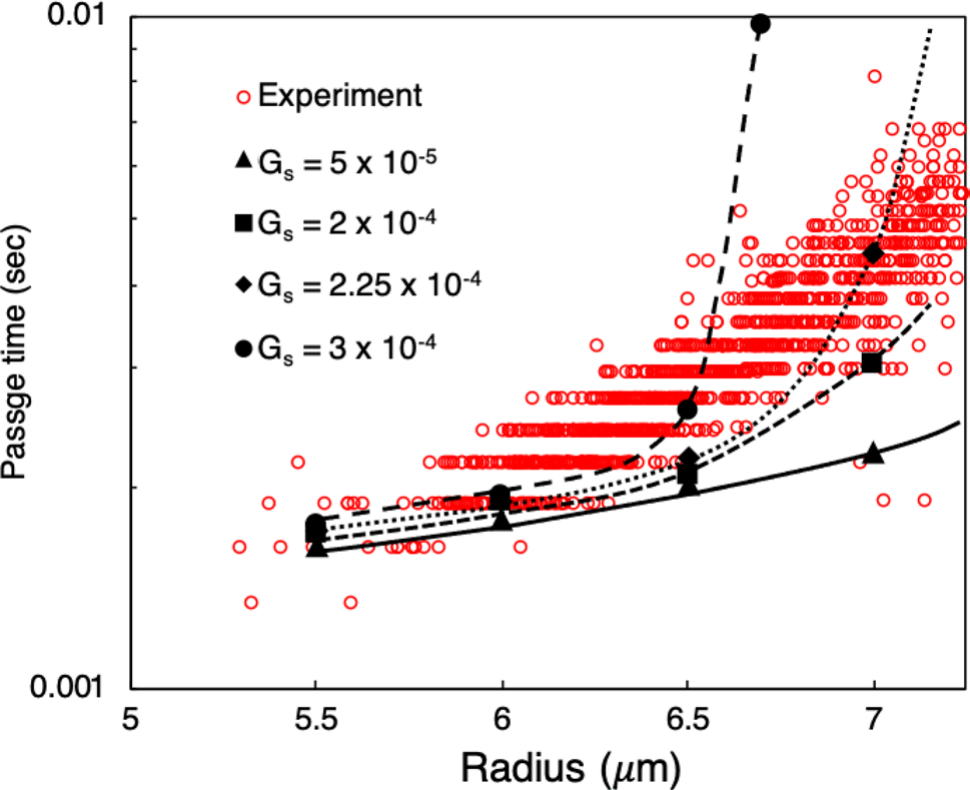
Passage times versus cell radius for simulated L1210 cells using the single-membrane model, compared with experimental data from Byun et al.^11^. Simulation data is given by the black data points for different $G_s$ values (N/m), and the experimental data is given by the open red circles.

Experimental data provided to us by the authors of Byun et al.^11^ is plotted in Fig. 5. This data gives the passage time for cells through the microfluidic constriction device as a function of cell size, with radii ranging from approximately $5.5\,\,\upmu \hbox {m}$ to $7\,\,\upmu \hbox {m}$. For our simulations we consider four different cell sizes to cover this range, and determine the passage time for each for different values of $G_s$.

For the smaller cell sizes, namely radii of $5.5\,\,\upmu \hbox {m}$ and $6\,\,\upmu \hbox {m}$, differences in the passage time for the given $G_s$ values are very small, and passage times agree well with the experiment as shown in Fig. 5. We observe that at these smaller sizes the cells can more easily deform to enter the constricted channel compared with the larger sized cells. This is evident from the similar passage times indicated by the data. While we observe a small decrease in cell velocity during entry, these sizes generally flow through the device with a similar average velocity to the background flow.

With increasing cell size larger discrepancies in passage time begin to manifest between the different $G_s$ values for a given cell size. These become significant as the cell radius increases to $7\,\,\upmu \hbox {m}$, and the results in Fig. 5 show the significant increase in sensitivity of passage time to $G_s$ for the larger sized cells. As can be seen, for a $G_s$ value of $3\times 10^{-4}\hbox {N/m}$ the cell passage time agrees for the $6.5\,\,\upmu \hbox {m}$ cell but significantly overpredicts it with increasing size. Conversely, for $G_s=2.25 \times 10^{-4}\,\,\hbox {N/m}$ the passage time agrees for the $7 \,\,\upmu \hbox {m}$ cell but underpredicts it for the $6.5 \,\,\upmu \hbox {m}$ cell. Overall, the general picture that arises from Fig. 5 is that for the smaller cells the single membrane model can agree with the data, but a single $G_s$ value cannot be found that results in good agreement for all cell sizes. For these experiments, the results for each cell size represent different degrees of deformation for cells from this line. These results thus suggest that the single-membrane model is sufficient to capture the transport behavior under circumstances limited to relatively small deformation.

With this type of modeling approach the cell resistance to deformation is localized to the outer membrane structure. In reality, with these types of cells this resistance is primarily caused by and distributed among the internal cell components as well. A number of works in the literature have developed models to represent these types of cells by considering an outer membrane structure with tensions on the order of $10^{-5}\,\hbox {N/m}$ enclosing a fluid significantly more viscous than the ambient^15,16,82,83^. With this approach the interior fluid is meant to collectively represent the behavior of the complex internal structure including cytoskeleton components. When modeled in this manner works have determined this viscosity based on a Newtonian model to range from the order of $10^1$ Pa-s ($10^4\,\hbox {cP}$)^82^ to $10^2$ Pa-s ($10^5\,\hbox {cP}$)^16,83^. Furthermore^19^, developed a slightly more complex representation of the inner fluid by considering it to be a shear-thinning, power-law fluid with a reference viscosity on the order of $10^2$ Pa-s ($10^5\,\hbox {cP}$).

In the present work we consider the fluid interior to the cell to be the same viscosity as the ambient fluid. With this, however, the results in Fig. 5 demonstrate that placing the cell resistance to deformation predominantly in the single membrane is insufficient to fully recreate the experimental data. With IBM-based approaches one can also model a viscosity contrast between the interior and exterior of the cell. However, the aforementioned viscosity values when representing the cell interior as a single-phase fluid are roughly five orders of magnitude higher than the water-like cytoplasmic fluid considered in the present work based on literature values^84^. This presents challenges with regard to numerical stability using our approach. As such, in the present work we do not explore this option, but instead in the subsequent sections focus on alternatives well suited to our simulation framework.

### Nucleated cell model accurately captures cell-specific transport behavior by modulating the nucleus stiffness ratio

Next, we improve upon the single membrane model and consider a cancer cell modeled by an outer plasma membrane and a nucleus membrane. This model is more phenomenologically representative of a cancer cell in that the resistance to deformation does not primarily reside in the outer membrane, but rather is distributed to an internal component as well. Furthermore, it is generally known that with such cells the nucleus membrane is much stiffer than the outer membrane^55,85–88^, a disparity that can be accounted for with this model. This is particularly important with cancer, because for situations in which cells undergo large deformations the stiffness of the nucleus can significantly influence the mechanical response^89^. In fact, experiments have shown that for situations in which these types of cells deform to move through narrow spaces, it is the nucleus that is the primary rate-limiting factor^90,91^.

**Figure 6:**
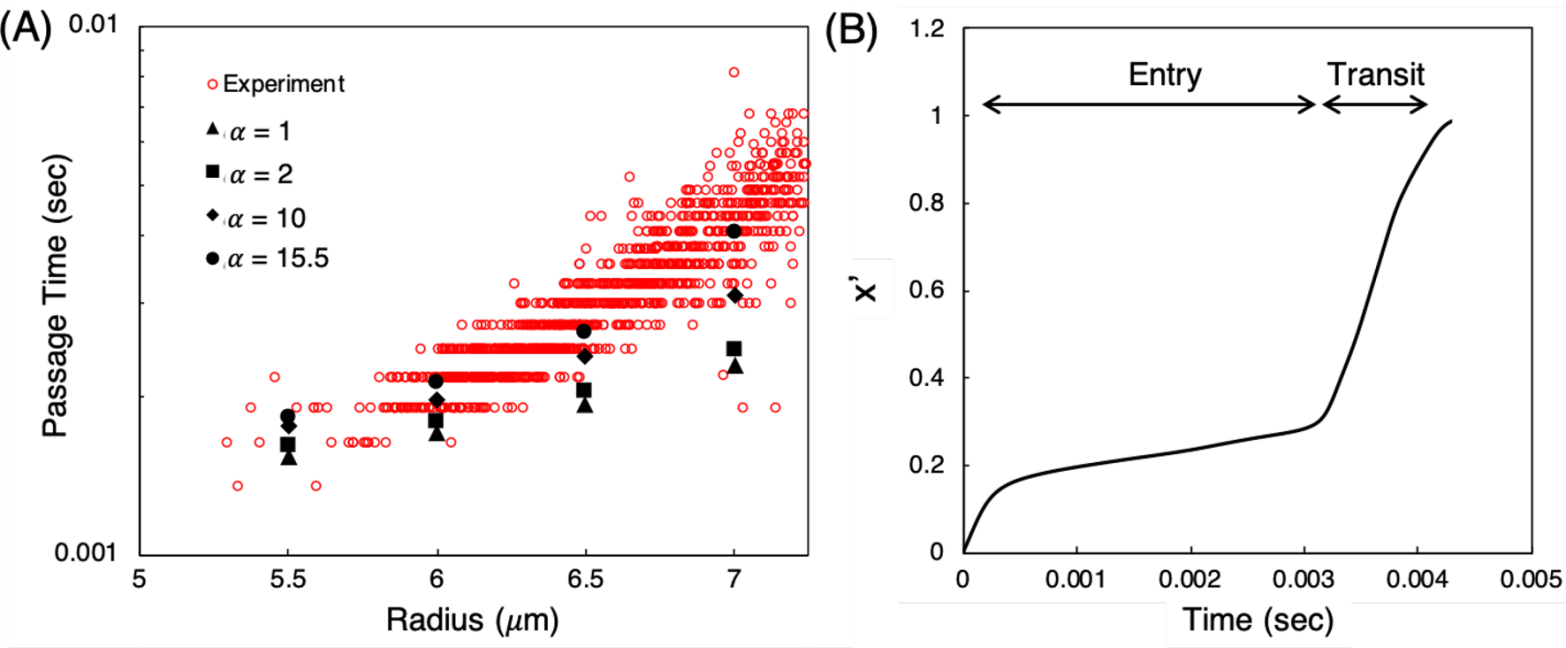
**(A)** Comparison between predicted passage times using the nucleated cell model (black data points) and experimental data (red data points) for L1210 cells^11^. With $\alpha =15.5$ the model gives good agreement with the data over the range of cell sizes. Differences in results between different $\alpha$ values also demonstrate the increased control over passage time by varying the nucleus stiffness when compared to the single-membrane model and Fig. 5. **(B)** Cell trajectory normalized by channel length ($x'=x/L$) versus time for the $\alpha =15.5$ case at $7\,\,\upmu \hbox {m}$, delineating the contributions of the cell entry and transit processes to the overall passage time.

With the nucleated cell model we take guidance from this insight provided by such experimental works and use the nucleus stiffness to modulate the cell passage time in our simulations. Specifically, we vary $G_s$ for the nucleus membrane and consider an outer membrane with $G_s=5\times 10^{-5}\,\,\hbox {N/m}$. We use this value because it is representative of that observed in experiments for plasma membrane stiffness with these types of cells^15,16,82,83^, as previously mentioned. Simulation results for cell passage time versus radius are provided in Fig. 6A along with the experimental data from Byun et al.^11^ for L1210 cells. Since we vary cell passage time by modulating the nucleus stiffness, the relevant parameter to consider is the ratio of $G_s$ values between the nucleus and outer membranes, or $\alpha =G_{s,nuc}$/$G_{s,outer}$.

The results in Fig. 6A show that with a nucleus membrane either equal to or twice as stiff as the outer membrane the predicted passage times generally fall below the data. However, increasing the stiffness ratio increases the passage time for each cell size to a degree resulting in these times approaching the experimental data. We determine that $\alpha =15.5$ results in cell passage times in agreement with the data over the range of cell sizes. Second, in Fig. 6B we plot the trajectory of the $7\,\,\upmu \hbox {m}$ cell for this ratio versus time, breaking down the observed passage time into entry and transit times. As can be seen, the entry process is much longer than the transit time through the constricted channel, which is caused by the reduction in cell velocity as it deforms to enter the constriction. Figure 6B also reflects the observed behavior that after the cell enters the constriction it rapidly transits through the channel. Generally speaking this relationship between entry and transit behavior was observed over the range of cell sizes considered, with differences between the two increasing with cell size. This is important to note because this behavior was also observed and noted in Byun et al.^11^. So while the results in Fig. 6A show agreement with the data for passage time, these observations on entry and transit times provide further qualitative validation with the experiments. They also complement the visual comparisons with the experiment provided in Figs. 3B,C.

#### Dependence of passage time on cell model input parameters to aid in characterizing cell types over a range of deformations

Another important observation from Fig. 6A is that with the nucleated cell model there is much better control over the cell passage time via the nucleus stiffness as compared with the single membrane model and the results in Fig. 5. We quantify this by looking at the sensitivity of changes in $\alpha$ to changes in passage time, and compare this with the sensitivity observed for the single membrane model as discussed in “Single-membrane model captures transport behavior under limited circumstances”. Specifically, for each cell size, we look at the increase in $G_s$ from one tested value to another by determining the ratio $\beta =G_{s,nuc}$/$G^0_{s,nuc}$. For this reference stiffness $G^0_{s,nuc}$ we use the smallest value considered, which is $5\times 10^{-5}\,\,\hbox {N/m}$ for the nucleated cell model. We also compute $\beta$ for the single membrane model, for which the reference stiffness is also $5\times 10^{-5}\,\,\hbox {N/m}$. To gauge the sensitivity of $\beta$ to passage time, we also compute the increase in passage time corresponding to the associated $\beta$ value, which we define as $\gamma =\tau _p$ / $\tau ^0_p$. Similarly, the reference passage time $\tau ^0_p$ is that which results from a cell with the reference stiffness.

**Figure 7:**
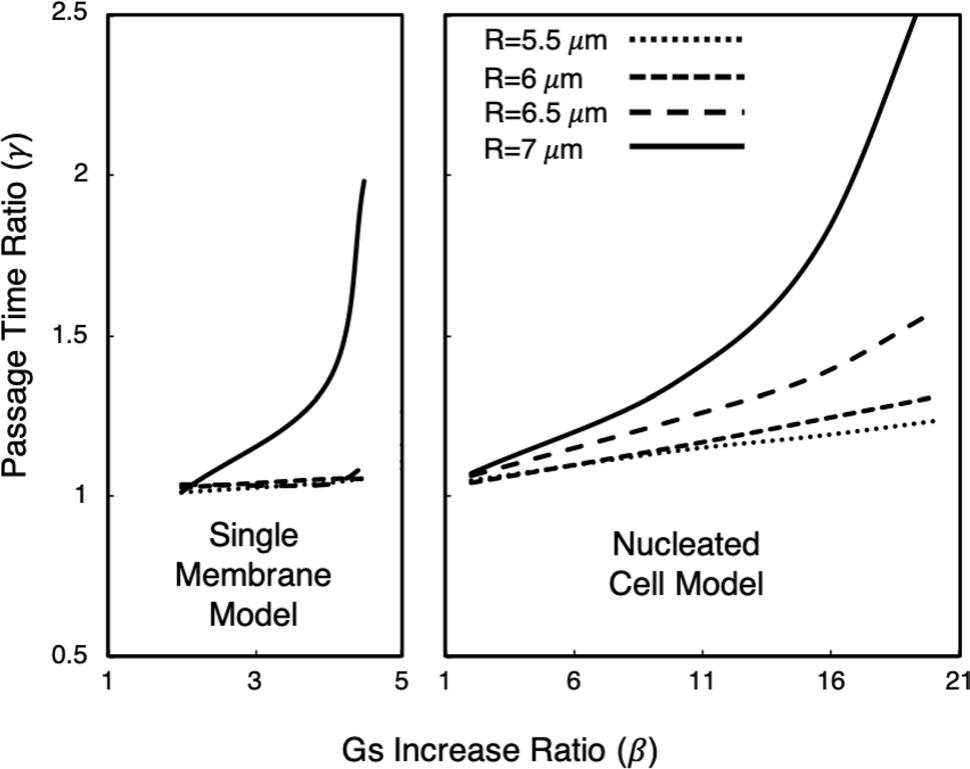
Relationship between increases in $G_s$ ($\beta$) and resulting increases in cell passage time ($\gamma$) for both the single-membrane and nucleated cell models, for each cell size considered. This illustrates the improvement in control over the passage time with the nucleated cell model. The trends for the nucleated cell model show that changing the nucleus stiffness causes a response in passage time to a relatively consistent degree. In contrast, the trends for the single-membrane model depict the comparatively poor control over the passage time.

In Fig. 7 we plot the relationship between $\beta$ and $\gamma$ for both the single-membrane and nucleated cell models. The improvement in control over the passage time is readily apparent from the non-negligible and relatively constant slope in the trends for the nucleated cell model. That is, this shows that changing the nucleus stiffness causes a response in the passage time to a relatively consistent degree. This is in contrast to the trends observed for the single membrane model. Figure 7 shows that for the 5.5–$6.5\,\,\upmu \hbox {m}$ cells the passage time minimally responds to changes in $G_s$. With the $7\,\,\upmu \hbox {m}$ cell there is a non-negligible slope up to $\beta \sim 4$, but then very abruptly increases. Collectively this observed behavior for the single-membrane model is not conducive to achieving good control over the passage time, and hence the observations in “Single-membrane model captures transport behavior under limited circumstances”.

With the cell models employed in the present work, we generally know a priori the critical model inputs on which we should focus in order to characterize the cell transport. This is based on what has been generally established and demonstrated in prior works involving both these types of models as well as experiments (e.g.^25,27,34,45,89–91^). More specifically, for the single membrane model the dominant input parameter controlling the outcome is the membrane stiffness, governed here by $G_s$. We note that there are other input parameters that will also influence the cell transport such as the driving pressure gradient, fluid properties, channel geometry, and cell size. However, in this work we are recreating specific experiments to interrogate the models against the data, which inherently limits the input parameters so that the conditions and specifications of the experiments are faithfully recreated. The primary goals here are then centered on how to tune the critical cell model inputs to match CTC data from these experiments in a way that can capture behavior to distinguish between different cell lines. For the non-nucleated cell model this allows us to narrow our focus to just $G_s$, and therefore the question becomes how sensitive is this model to changes in $G_s$ which is addressed by Fig. 5 and complemented by Fig. 7 above (left-most panel). Furthermore, the sensitivity of passage time to cell size is a dependency specified by the data, and physiologically the cell properties (i.e. $G_s$ with this model) should not be dependent on cell size. Hence, quantifying passage time sensitivity to $G_s$ in the manner of Fig. 5 provides a physiologically consistent means of illustrating the critical dependencies with this model.

For the nucleated cell model, the critical input is not just $G_s$ but rather the nucleus stiffness ratio (i.e. $\alpha$ in “Nucleated cell model accurately captures cell-specific transport behavior by modulating the nucleus stiffness ratio” and Fig. 6) as well as the nucleus size. We know a priori to focus on these inputs for this model since, as mentioned, it has been established by prior works that the nucleus stiffness is the dominant physiological cell characteristic which influences transport behavior during passage through small openings^89–91^. The size of the nucleus will naturally affect the degree to which its stiffness modulates passage behavior, and for situations where $\phi$ is not known the sensitivity to that parameter is also important. For the nucleated model then, this allows us to narrow our focus to $\alpha$, and if needed $\phi$, and therefore the question becomes how sensitive is passage time to these two critical parameters. For the L1210 line, $\phi$ is known and so we focus our analysis here on sensitivity to $\alpha$. For the lung cancer line presented later in “A systematic approach to model a different cell line using the nucleated cell model”, both $\alpha$ and $\phi$ are not known, and the analysis therein extends the sensitivity of passage time to both of these inputs.

Overall, the results and analyses associated with Figs. 5, 6 and 7 reveal that using the nucleus as the primary rate-limiting factor results in much better control over the passage time than the single-membrane model. With the single membrane model the resistance to deformation is placed solely in the outer membrane which is a much larger structure than the nucleus. Here we show that by distributing the resistance among different cell components, which is more phenomenologically realistic, we can determine cell properties to fit the experimental data over a range of cell sizes. Another perspective is that since the channel size is fixed, the cases for each cell radius correspond to situations in which the degree of cell deformation varies. Thus, these findings also suggest that for situations in which cell deformation is relatively small, the single membrane model may be sufficient to capture cell behavior, but with increasing deformation the nucleated cell model is needed.

#### Nucleated cell model reproduces behavior from a separate experiment, verifying cell properties and expanding the range of application

With the nucleated cell model sufficient to match the experimental data from Byun et al.^11^, we recreate a separate experiment^54^ to verify the L1210 cell properties determined for this model in “Nucleated cell model accurately captures cell-specific transport behavior by modulating the nucleus stiffness ratio”. We also use this to verify our approach for recreating the experiments of Byun et al.^11^. This is done with an eye towards also being able to use data from^11^ to model other types of cancer cells using the nucleated cell model (“A systematic approach to model a different cell line using the nucleated cell model”).

The experiments in Guo et al.^54^ involved L1210 cells deforming through a microfluidic ratchet device as detailed in “Recreating a separate experiment to verify cell properties and interrogate model accuracy under different conditions”. The nature of these experiments is somewhat different than Byun et al.^11^ in that here the metric gauging deformability is not time-based. That is, the experiments here involved determining the minimum pressure required to drive a cell through, and did not involve the rate at which the cell passed through as was the case in^11^. In this sense this experiment provides an additional perspective from which to investigate this cell model. As discussed in the Introduction, this consideration is important because it establishes the accuracy in capturing not only the more dynamic behavior as in^11^, but also the much more gradual large deformation behavior observed in this experiment. Microcirculatory CTC transport exposes CTCs to a range of conditions, and thus interrogating the model using both of these experiments provides for a more robust validation to expand the range of application.

**Figure 8:**
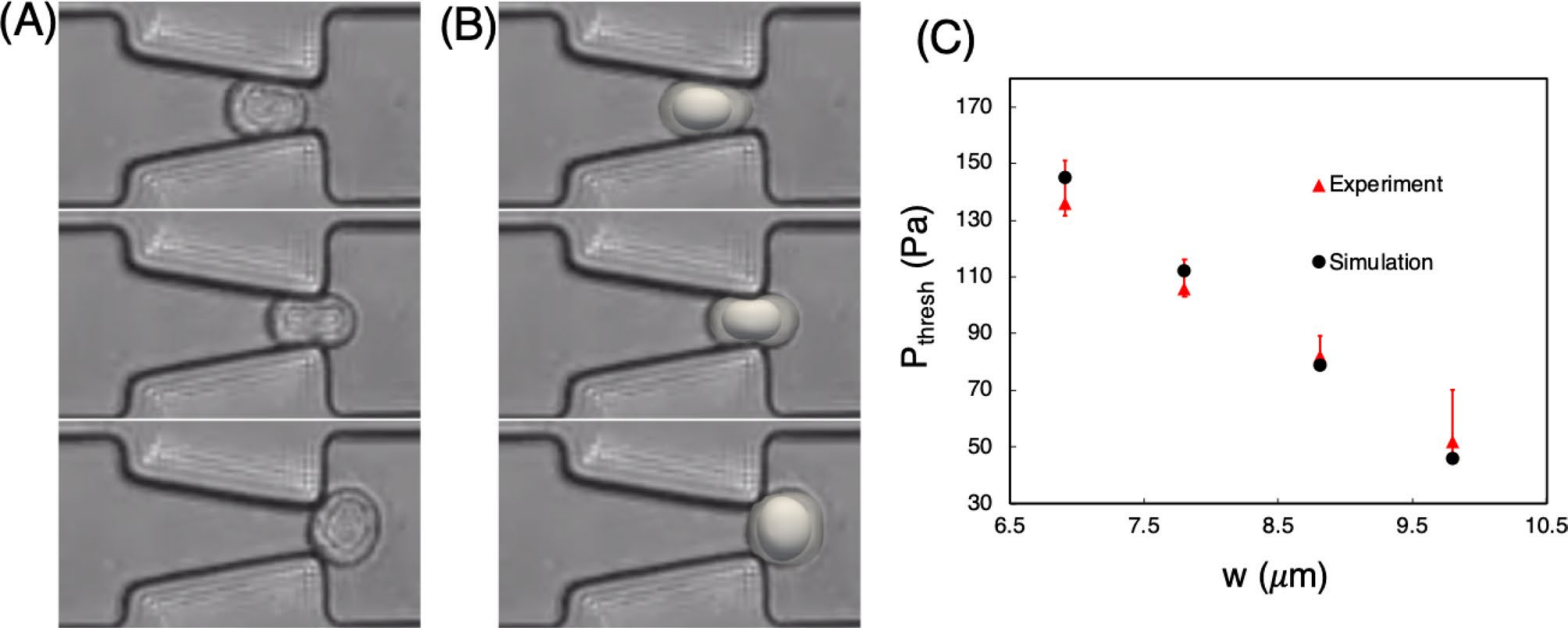
Comparisons between experiments^54^ and simulations using the nucleated cell model. **(A)** Experimental images of an L1210 cell at different locations as it squeezes to pass through the opening. In these figures the cell moves from left to right. Images are taken from a video in the supporting information of^54^. **(B)** Snapshots of a simulated cell superimposed on the experimental images in **(A)** at the same locations as in the experiment. **(C)** Threshold pressure ($P_{thresh}$) versus pore size (w), with experimental values taken from^54^ given by the red triangles and data range given by the vertical lines. Simulation values are given by the closed black circles.

Qualitative and quantitative comparisons between our simulations and the experiments are provided in Fig. 8. In Fig. 8A we present images taken from a movie included with Guo et al.^54^, depicting an L1210 cell at different locations as it squeezes to pass through the opening. We compare the qualitative behavior of our simulated cell with this in Fig. 8B, showing shapshots of our nucleated cell model superimposed on the experimental images at the same locations as in the experiment. As is evident, the cell shapes agree well between the two for the shapes observed in each of the frames. In the top frame the cells take on a tapered shape, which transitions to a more pinched shape in the middle frame as the cell passes through the opening. The bottom frame gives the cell shape after it has passed through the opening, where it can be seen that the cell relaxes into a more spherical shape. The shape of the nucleus can also be seen in the experimental images during this process, and are included with the simulated cell snapshots as well. In the first two frames the location of the nucleus in the experiment appears closer to the leading edge of the outer membrane than in the simulation. Aside from this, the shapes qualitatively appear to be in general agreement with one another.

In Fig. 8C we present quantitative comparisons with the experiment by plotting the $P_{thresh}$ value versus pore size (w). As can be seen, for each pore size the threshold pressure value determined by the simulations is within the range of values reported in Guo et al.^54^. The smallest pore size of $6.9\,\,\upmu \hbox {m}$ represents the largest degree of cell deformation and thus the largest required $P_{thresh}$. With our model we determine $P_{thresh}=145\,\,\hbox {Pa}$, while the mean value from the experimental data is approximately 136 Pa, and ranged from roughly 131 Pa to 151 Pa. With increasing pore size, as shown in Fig. 8C the required $P_{thresh}$ value decreases, as would be expected. For the $7.8\,\,\upmu \hbox {m}$, $8.1\,\,\upmu \hbox {m}$ and $9.8\,\,\upmu \hbox {m}$ cases we determine $P_{thresh}$ values of 112 Pa, 79 Pa, and 46 Pa, respectively. These match well with the mean values from the experiments, and as mentioned are within the range of values reported.

Overall, these results quantitatively verify both the L1210 properties determined using data from Byun et al.^11^ as well as our approach to recreate the experiments performed therein. They also provide a further validation of the nucleated cell model itself and its ability to reproduce large deformation behavior observed in experiments over a range of conditions.

### Adaptive cyto- and nucleo-skeleton components capture large deformation behavior with same accuracy as nucleated cell model

We also investigate a nucleated cell model augmented by cyto- and nucleo-skeleton components. While the nucleated cell model in “Nucleated cell model accurately captures cell-specific transport behavior by modulating the nucleus stiffness ratio” was shown to be sufficient to reproduce experimental data, the membrane resistance to deformation is predominantly in-plane. Thus, here we briefly discuss matching experimental data with this augmented approach. This offers an improved representation of the mechanical cell structure over that in “Nucleated cell model accurately captures cell-specific transport behavior by modulating the nucleus stiffness ratio” by modeling components internal to the membranes which also contribute to deformation resistance. This can potentially be used to model inhomogeneities in cell properties by modulating individual spring properties, or adapting it to model cell division similar to what has been done in other works (e.g.^24^).

**Figure 9:**
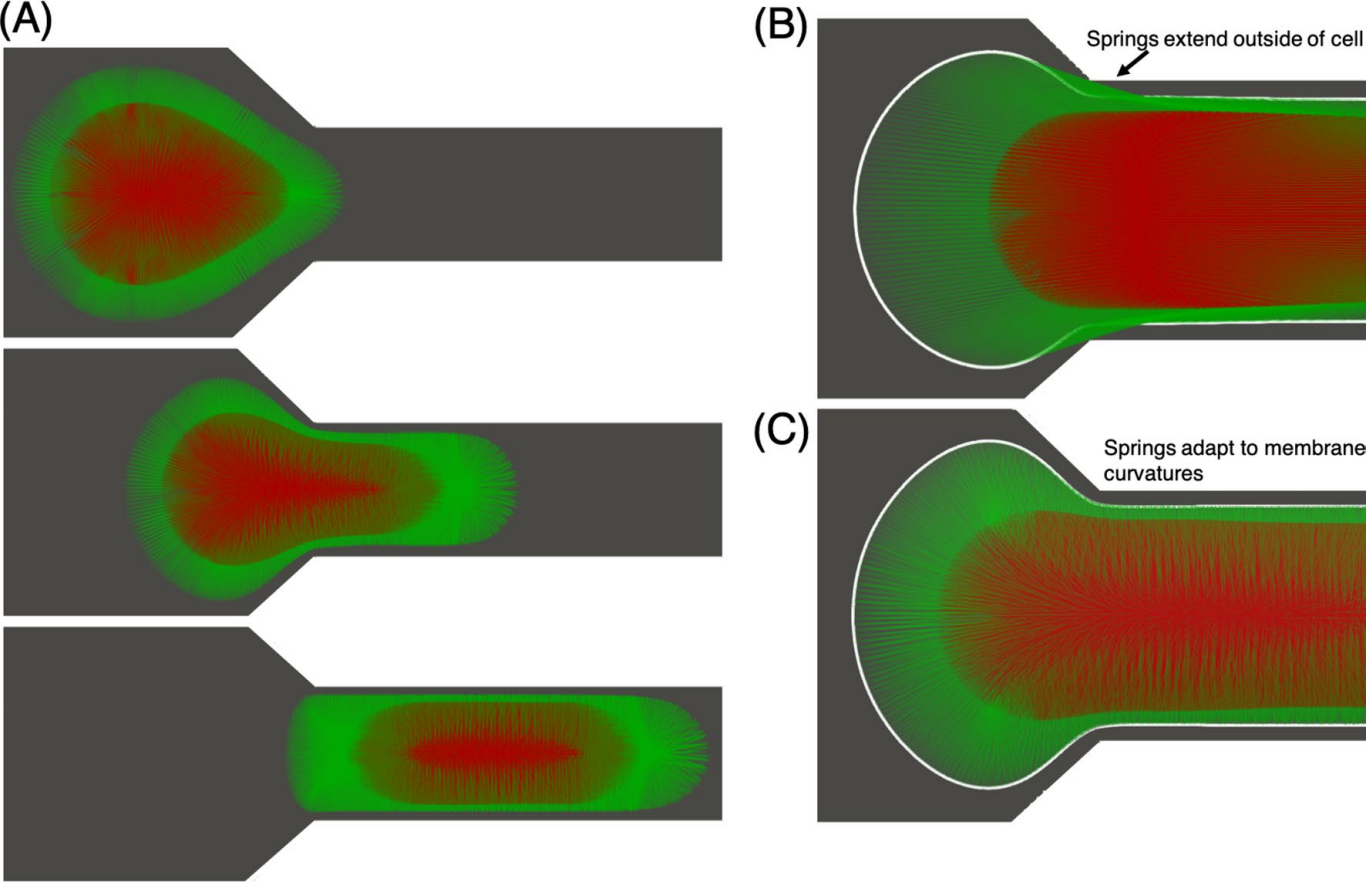
Simulation snapshots depicting the nucleated cell model augmented by cyto- and nucleo-skeleton components deforming to enter the constricted microchannel. Cytoskeleton springs are given by green lines, and nucleoskeleton springs are given by red lines. **(A)** Snapshots in sequence showing the deformation associated with the process of cell entry, and the adapting structure of the springs. **(B,C)** Zoomed in view depicting the end of the entry process. In **(B)** we show how springs can extend outside of the cell due to the instantaneous membrane structures, which is remedied by implementing an adaptive technique **(C)** where the springs restructure based on the membrane curvatures.

Figure 9A provides snapshots of a simulated cell deforming to enter the constricted microchannel. The cytoskeleton springs are given by the green lines and the nucleoskeleton springs are given by the red lines. For simplicity we consider each spring to have the same Young’s modulus, and we modulate the cell deformability using this quantity. As with the previous section we consider an outer membrane with $G_s=5\times 10^{-5}\,\,\hbox {N/m}$, and here consider a nucleus membrane with a representative stiffness ten times greater than the outer one. We find that a Young’s modulus of 950 Pa gives passage times in agreement with the experimental data in Fig. 6A to a similar degree of accuracy as with the nucleated cell model. We note that, given the results from the nucleated cell model in “Nucleated cell model accurately captures cell-specific transport behavior by modulating the nucleus stiffness ratio”, the purpose of this section is simply to demonstrate the basic features of this model and its ability to recreate experimental data.

An important modeling consideration with this spring-based approach is its ability to accommodate complex deformations. The initial structure of the cytoskeleton springs connecting the outer membrane to the nucleus membrane is determined by projecting the centroid of each triangular element of the surface mesh for the outer membrane onto an element of the nucleus membrane. Similarly, for the nucleoskelton springs the centroid of each element on the surface of the nucleus membrane is projected inward and connected to the element on the opposing side. This provides a convenient framework for initializing the cyto- and nucleoskeleton structures. However, what we observe for the present simulations is that maintaining this initial spring network can result in temporary unphysical behavior where the springs extend outside of the cell. This occurs due to the temporary concavity of the membranes resulting from the complex deformation which the cell experiences for this particular geometry. An example of this behavior is shown in Fig. 9B. To overcome this we implement a technique where springs adapt to the instantaneous cell curvatures, as discussed in “Using an adaptive spring-based approach to model the cyto- and nucleo-skeleton components”. An example of this is shown in Fig. 9C. Compared with the behavior in Fig. 9B, the springs do not extend outside of the membrane and instead restructure in a manner specifically tailored to the instantaneous cell shapes. This adaptive technique thus provides a robust modeling approach which permits such complex deformations. Furthermore it ensures that springs exist within all regions of the cytoplasm as its shape evolves with time.

### A systematic approach to model a different cell line using the nucleated cell model

Lastly, we use the nucleated cell model to reproduce experimental data for a murine lung cancer cell line^11^. This model was shown to be sufficient to accurately capture the behavior of L1210 cells, and here we focus on a different line in order to both interrogate the adaptability of this model as well as broaden the range of potential applications.

Following our approach in “Nucleated cell model accurately captures cell-specific transport behavior by modulating the nucleus stiffness ratio”, we consider a representative value for the outer membrane stiffness of $G_s=5\times 10^{-5}\,\,\hbox {N/m}$, and use the nucleus stiffness to modulate the cell passage time. We consider four different cell sizes over the range of radii from $5.5\,\,\upmu$m to $7\,\,\upmu$m, and we compare passage times from our model against data given to us by the authors of^11^. Regarding the nucleus size, for the L1210 cells we used values reported in the literature, however for this cell line the nucleus size was not known a priori. Thus, we consider it here as an additional unknown. For this cell line, tuning the model to match the experimental data then involves determining the influence of both nucleus size and nucleus stiffness on the cell passage time.

**Figure 10:**
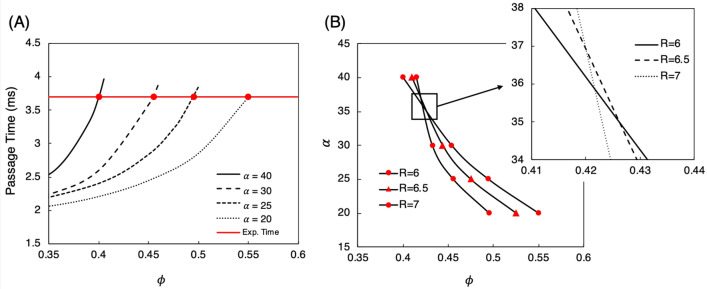
Determining the dependency of passage time on $\phi$ (karyoplasmic ratio) and $\alpha$ (nucleus membrane stiffness ratio). **(A)** Results for $\phi$ vs. passage time for the $6\,\,\upmu$m cell. The four black curves correspond to different $\alpha$ values, and the red line gives the passage time centered on the experimental data. The closed red circles give the $\phi$ values which result in the experimental passage time for the respective $\alpha$ value. This determined $\phi -\alpha$ relationship for the 6 $\upmu$m cell is then plotted in **(B)** along with the relationships determined in the same manner for the 6.5 and $7\,\,\upmu$m cells. The inset here shows the region where the curves overlap. Although they do not all intersect at one point, the region centered on $\phi \sim 0.425$, $\alpha \sim 35.5$ represents the optimal values to yield passage times centered on the data for each of these cells sizes.

We quantify the nucleus stiffness as we did in “Nucleated cell model accurately captures cell-specific transport behavior by modulating the nucleus stiffness ratio” using the stiffness ratio $\alpha$, and we quantify the nucleus size using the karyoplasmic ratio, $\phi$, which gives the ratio of nucleus volume to cell volume. To gain insight into the dependency of passage time on these quantities, we first consider a cell with radius of $6\,\,\upmu m$ and determine $\phi$ versus passage time for a given $\alpha$. We choose this size as it is near the middle of the sizes considered. In Fig. 10A we plot results for four different $\alpha$ values ranging from 20 to 40. Although we tested $\alpha$ values above and below this range, we limit the results shown here to more clearly illustrate the processes of fitting to the experimental data. We also plot the passage time centered on the experimental data for this size cell, which is approximately 3.7 ms.

We observe in Fig. 10A that the passage time exponentially increases with increasing $\phi$ for each of the values considered, although at different rates for each $\alpha$. For the smallest nuclear stiffness ($\alpha =20$) the increase in passage time with increasing $\phi$ is more gradual compared to the other cases. Furthermore, between each $\alpha$ in sequence the rate progressively increases. Of interest here is specifically the $\phi$ value resulting in a passage time centered on the experimental data, which is different for each $\alpha$ value. To illustrate this, also shown in Fig. 10A is the point of intersection for each $\alpha$ curve with the desired passage time, which gives this corresponding $\phi$ value. Using the $\alpha$ and $\phi$ values corresponding to each intersection point we plot the relationship between these two quantities in Fig. 10B to illustrate the dependence of $\alpha$ on $\phi$ required to fit the experimental passage time for the $6\,\,\upmu$m radius cell. This shows the observed trend that with increasing nucleus size the nucleus stiffness required to fit the experimental passage time decreases, which is intuitive and expected. As can be seen, however, we observe this relationship to be non-linear; there is an abrupt drop in the required $\alpha$ with increasing $\phi$ up to approximately 0.45, after which $\alpha$ decreases at a slower rate.

**Figure 11:**
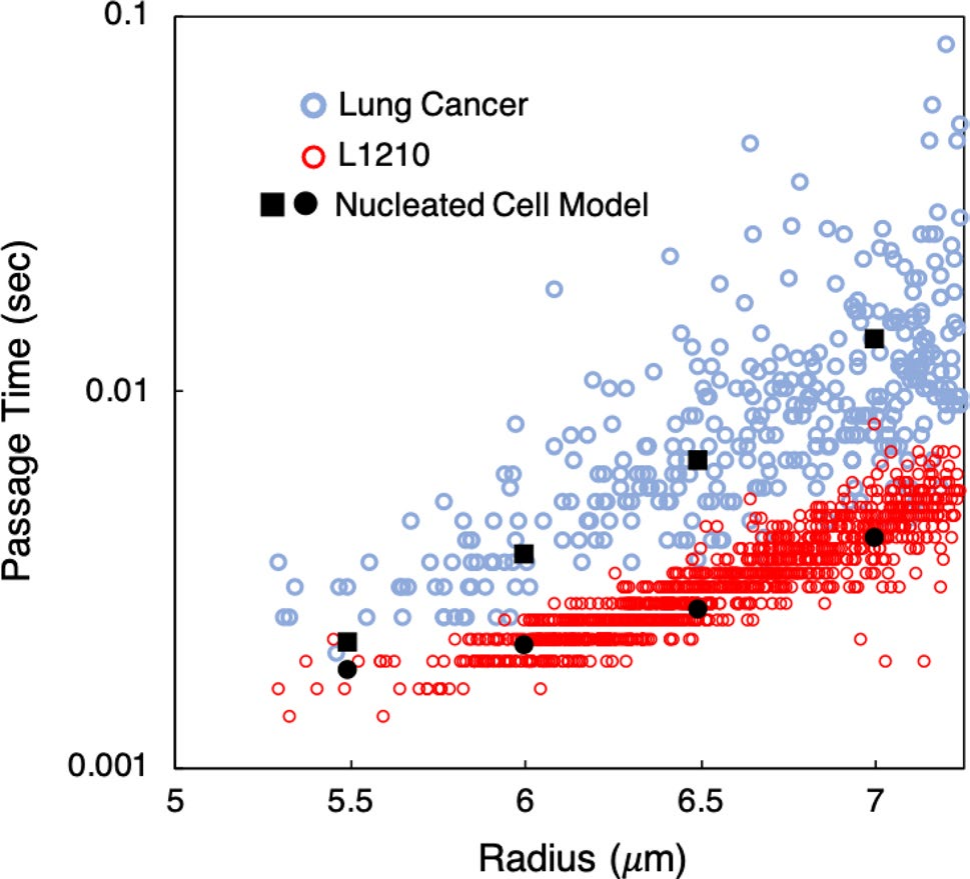
Passage time versus cell size for murine lung cancer cells, in addition to L1210 cells. Experimental data from the authors of^11^ are given by the blue and red data points for the respective cell lines. For the lung cancer line, the passage times determined using the nucleated cell model with $\alpha =35.5$, $\phi =0.425$ are given by the black squares. Data for L1210 cells is also included from Fig. 6A for $\alpha =15.5$, to highlight the versatility of the model to capture behavior that can distinguish between cell lines.

Next, we repeat this process for $6.5\,\,\upmu$m and $7\,\upmu$m radii cells using the same $\alpha$ values, with the goal of achieving the average experimental passage times of approximately 6.5 ms and 12 ms, respectively. In Fig. 10B we plot the relationship between $\alpha$ and $\phi$ determined for these cells as well. As can be seen, the general trend is similar to that observed for the $6\mu m$ cell in that there is a non-linear relationship observed between $\alpha$ and $\phi$. However, the initial decrease in $\alpha$ is increasingly abrupt with increasing cell size. This occurs because the $\phi$ value determined to fit the respective passage times for $\alpha =40$ increases with decreasing cell size, but for $\alpha =30$ the trend is opposite. While we do not observe these curves to intersect at one ($\alpha ,\phi$) location, the inset to Fig. 10B shows a general region centered on $\alpha \sim 35.5$, $\phi \sim 0.425$ where the curves are closest to one another. This location is important because it represents the optimal combination of nucleus size and stiffness values that give passage times in reasonable agreement with the data for each cell size. Using these values then we plot the passage time over the range of cell sizes in Fig. 11 against the experimental data. As can be seen, the times are in good agreement with the data for each cell size. We also include in this figure the data for L1210 cells from Fig. 6A for $\alpha =15.5$, to further highlight the versatility of the model to capture distinctly different cell behavior that can distinguish between cell lines.

## Conclusions

Through detailed comparisons with experiments, we have elucidated a means of using a membrane model-based approach to reproduce data for cancer cells from specific lines undergoing large deformation. We have considered single-membrane, nucleated cell, and cyto- and nucleo-skeleton models in an IBM-based in silico framework. Experiments were recreated in which murine leukemia cells and lung cancer cells squeeze to pass through a microfluidic constriction. Using this, we have provided new insights into the comparative behaviors of each model and their suitability to reproduce relevant behavior.

The following summarizes the main findings of this work:

1. We find that the single-membrane model is able to reproduce experimental data^11^ under limited circumstances for L1210 cells. For the smallest cells considered a membrane stiffness can be determined to yield reasonable agreement with the data for passage time. However, for larger cells the results deviate from the data. As the cell size here varies but the geometry remains fixed, these findings suggest that for situations in which cell deformation is relatively small the single membrane model may be sufficient. With increasing deformation, however, a more complex model seems warranted.
2. For the nucleated cell model we find that using the nucleus stiffness to modulate overall cell deformability gives much better control over the passage time through the microchannel than with the single-membrane model. As a result, we are able to determine stiffness values using this model to reproduce experimental data^11^ for L1210 cells over the range of cell sizes. This demonstrates that the nucleated cell model is sufficient to recreate the passage of L1210 cells squeezing through the microfluidic constriction.
3. We have compared the nucleated cell model with data from a separate experiment involving L1210 cells^54^ in which the cell size was fixed but the channel size varied. We observe good agreement with this other data over the range of channel sizes. This provides a verification of the cell properties determined using data from^11^ as well as our approach to recreate the experiments performed therein.
4. Using data from^11^ we have shown that the nucleated cell model can also be used to recreate the passage of murine lung cancer cells through the microfluidic constriction. By parametrically varying both the nucleus size and stiffness, a single combination was determined which results in passage times in agreement with the data over the range of cell sizes. In so doing we have outlined a systematic approach to tune this model to represent different cell lines.
5. We have briefly discussed a nucleated cell model augmented by springs meant to mimic the mechanical structure of the cyto- and nucleo-skeleton components. We have presented an adaptive technique whereby springs restructure to instantaneous membrane curvatures, which permits the modeling of complex deformations with such an approach. This model was found to yield similar accuracy to the nucleated cell model, and as such was not required here to reproduce the experimental data and relevant behavior. However, this can be used to capture more complex processes not considered here such as cell division, or to model cell inhomogeneities if warranted, among others.

To our knowledge this is the first work to interrogate cancer cells modeled with Skalak’s law against experimental data for different types of cancer. Our findings provide evidence that this IBM-based approach can yield good agreement with experimental data for cancer cells undergoing large deformation. We have also shown how it can be used to capture differences in cell behavior to distinguish between different cancer cell lines. Owing to the demonstrated accuracy and the versatility of this approach, going forward this can be used to model specific cancer cell lines in cell-resolved blood flow simulations through the microcirculation. This offers the potential to provide new insights into the hemodynamic mechanisms underlying the spread of cancer, and better understand characteristics unique to different cancer types.

## Supplementary Information

Supplementary Information.
