# Supplementary material for: A data-driven approach to modeling cancer cell mechanics during microcirculatory transport
Source: Sci Rep. 2021 Jul 27;11:15232. doi: 10.1038/s41598-021-94445-5 (PMC8316468; doi:10.1038/s41598-021-94445-5)
Supplement: Supplementary file 1 — Supplementary Information. [file 41598_2021_94445_MOESM1_ESM.pdf]

Supplementary Information: A Data-Driven Approach to Modeling Cancer Cell Mechanics During Microcirculatory Transport

Peter Balogh[1], John Gounley[2], Sayan Roychowdhury[1], and Amanda Randles*[1]

[1]Duke University, Department of Biomedical Engineering, Durham, NC
[2]Oak Ridge National Laboratory, Computational Sciences and Engineering, Oak Ridge, TN
*amanda.randles@duke.edu

1 Multi-resolution modeling approach

Here we detail our multi-resolution approach to recreate the experiments of *Byun et al.*[1]. What we've termed as the full model in the main text is given in Figure 2A, with the corresponding multi-resolution approach depicted in Figure 2B. For the full model, the fixed pressure boundary conditions are applied at locations 1 and 5, with values determined based on hydraulic calculations for the experimental setup described in *Byun et al.*[1]. The multi-resolution model uses these fixed values in conjunction with other relevant quantities discussed below, and dynamically determines the pressure to be applied at the inlet to the 3D model, denoted as point A in Figure 2B.

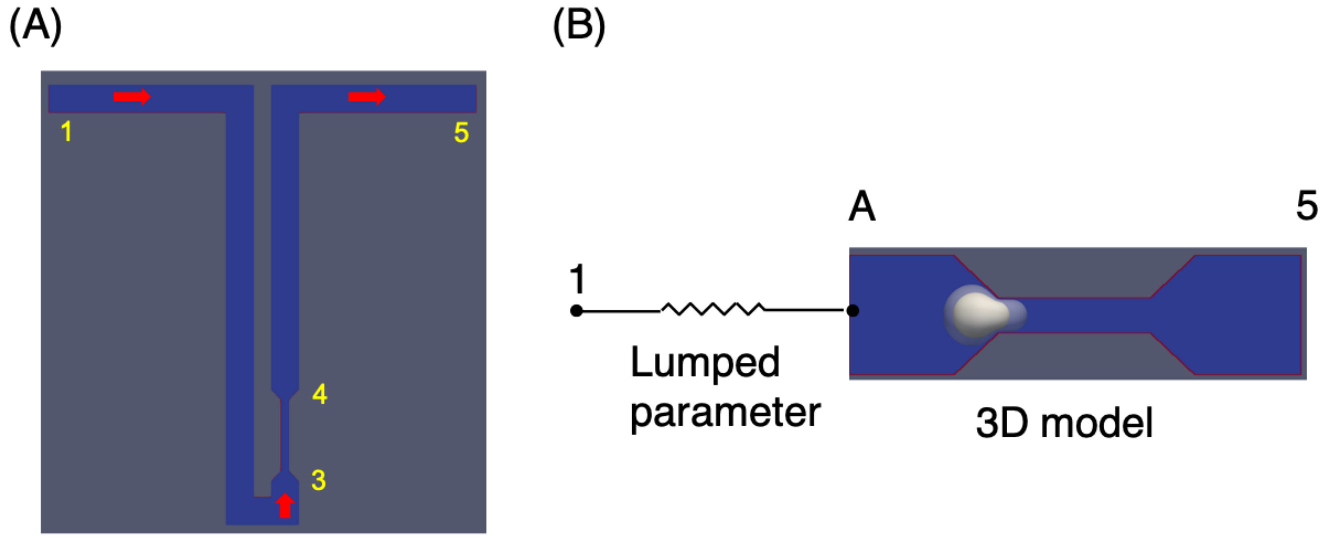

Figure 1. Schematic depicting each of the models. (A) Provides the full model, where the red arrows give the flow direction.(B) The multi-resolution model, where half of the constriction region and the vessels upstream and downstream are modeled with a lumped parameter approach, and the full 3D model is used for the relevant region of interest that captures cell entry and transit through the constriction.

With the full model, the constriction region between locations 3 and 4 is 50μm in length, with cross-section dimensions of 6μm x 15μm. We consider the channel length upstream and downstream of this to be 1000μm, with cross-section dimensions of 20μm x 15μm. As discussed in the main text, with our multi-resolution approach we model a portion of this as fully resolved in 3D, and the remainder with a lumped parameter model. The 3D model

consists of a constriction region that is 25μm in length, and inlet and outlet regions totaling 50μm in length. The remaining channel lengths, namely 950μm for the main channel and 25μm for the constriction region, are modeled with the lumped parameter model.

The procedure for determining the pressure value to be imposed for the 3D model (P_A, corresponding to location A in Fig. 2B) is described below, and is applied at each timestep of the simulation. P_A is determined based on the flow rate determined by the 3D model after completing a timestep, however simply applying this P_A value as as the boundary condition at the next timestep resulted in severe instabilities. To remedy this, we utilized a proportional-integral (PI) controller[2] to determine the value to be imposed.

Procedure:

1. Impose P_A and run a 3D simulation time step
2. Extract the flow rate (Q) from the 3D simulation
3. Determine the pressure drop through the lumped parameter model, ΔP_{lumped}:

$$\Delta P_{lumped} = G_{main} \cdot L_{main} + G_{constr} \cdot L_{constr} \tag{1}$$

   where the *main* and *constr* subscripts refer to the main and constriction regions of the channel, respectively. G is the pressure gradient along the channel, and is computed based on the flow rate Q from Boussinesq's solution[3] for pressure driven flow through a rectangular channel with cross-section h x l:

$$G = Q\left[\frac{h^3 l}{12\mu} - \frac{16h^4}{\pi^5\mu}\sum_{n=1}^{\infty}\frac{1}{(2n-1)^5}\frac{\cosh(\beta_n l)-1}{\sinh(\beta_n l)}\right]^{-1} \tag{2}$$

   where μ is the dynamic fluid viscosity, and $\beta_n = (2n-1)\pi/h$.

4. Determine an intermediate pressure:

$$P_A^* = P_1 - \Delta P_{lumped} \tag{3}$$

5. Define the difference between this and the current value as the error:

$$P_{err} = P_A^* - P_A \tag{4}$$

6. Feed this into the PI controller, and determine ΔP_A:

$$\begin{aligned} \alpha_p &= P_{err} \cdot K_p \\ \alpha_i &= P_{err} \cdot K_i \cdot /60 + \alpha_i \\ \Delta P_A &= \alpha_p + \alpha_i \end{aligned} \tag{5}$$

   where the proportional and integral gains used are $K_p = 0.1$ and $K_i = 0.01$, respectively.

7. Determine the new P_A to be imposed at the next time step:

$$P_A = P_A + \Delta P \tag{6}$$

To verify that the multi-resolution model gives the same result as the full model, the cell passage time through the constriction as predicted by each is compared. To illustrate this, in Figure 3 we plot the cell centroid position versus time for a representative cell. As can be seen, the trajectories agree well with one another, thus verifying the multi-resolution model. We note that the passage time predicted by the multi-resolution model is the sum of cell entry time and cell transit time through the constriction region. With our approach here where only half of the cell transit through the constriction region is modeled, we determine the cell transit time by doubling the value determined from our model. Preliminary testing involving comparisons between the full and multi-resolution models has verified consistent results.

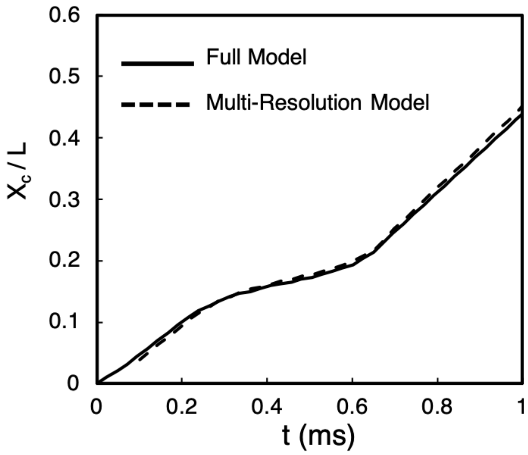

Figure 2. Comparison between the full and multi-resolution models. Here we plot the trajectory of a representative cell entering and transiting through the constriction. The y-axis gives the position of the cell centroid normalized by the length of the constriction region. The decrease in cell velocity as it deforms to enter the constriction is evident from the change in slope between approximately 0.25 and 0.5ms. As can be seen, the two models agree well with one another, thus validating the multi-resolution model.

2 Mesh refinement studies

Here we detail mesh refinement studies performed to determine the required model resolutions to sufficiently capture the relevant physics. With the immersed boundary method (IBM)-based approached employed in the present work, model resolutions refer to both the fluid lattice resolution for the LBM solver, as well as the FEM mesh resolution for the cell solver.

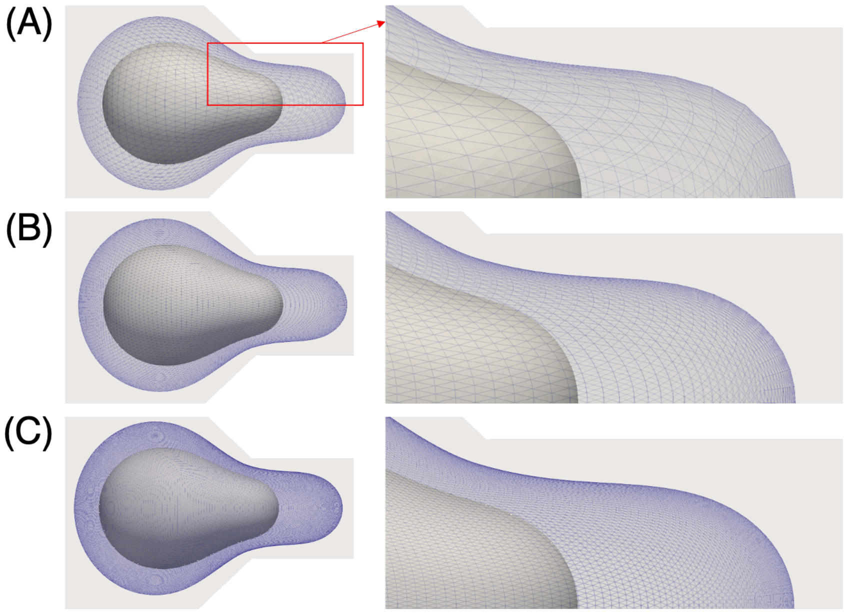

Figure 3. FEM mesh resolutions considered for the refinement studies, shown for representative nucleated cells deforming to enter the microfluidic constriction. The meshes depicted in Figures A-C correspond to those with 5120, 20480, and 81920 elements, respectively, generated by successively refining an icosahedron 4, 5, and 6 times. For each figure, the inset provides a zoomed-in view of the deformed cell within the constriction, with the outer membrane mesh given in blue, and the nucleus given by the solid grey.

For the LBM-based fluid solver, the lattice spacing must be sub-micron given the cell diameters are on the order of 10μm. Additionally, we know that the microfluidic constriction through which the cells traverse has a height of 6um. Previous work in a similar geometry[4] has shown that a fluid resolution of 0.15um is sufficient to capture the fluid dynamics, which we verify here for the present simulation setup by considering resolutions of 0.2, 0.15, and 0.1μm. For the FEM-based cell solver, the surface mesh comprised of triangular elements is generated by successively refining an icosahedron. Here we consider refinement levels of 4, 5, and 6, which correspond to 5120, 20480, and 81920 elements, respectively. Representative examples of the IBM cell and LBM fluid meshes at these resolutions are provided in Figures 3 and 4.

Simulations are performed at each of the aforementioned resolutions using the nucleated cell model, considering a representative cell with a radius of 6μm, an outer membrane shear elastic modulus of G_s=5x10^{-5}N/m, and a nucleus shear elastic modulus of G_s=1x10^{-4}N/m. We model the passage of this cell through the microfluidic constriction device of *Byun et al.*[1] driven by a fixed pressure drop, and given the above resolutions and the different combinations thereof results in nine cases. The primary model output for the present work which we are using to compare against experimental data is the cell passage time, and for each of the cases we plot this in Figure 5. As can be seen, the passage times predicted with a fluid resolution of 0.2um are consistently above that predicted at the other fluid resolutions for each of the FEM mesh resolutions. That is, at this fluid resolution increasing the FEM mesh resolution alone is not sufficient to achieve convergence with respect to the passage time. Increasing the fluid resolution to 0.15um and comparing passage times to that predicted at 0.1um resolution, the curves can be observed to overlap within increasing FEM mesh resolution. At an FEM mesh resolution of 5120 there is a very

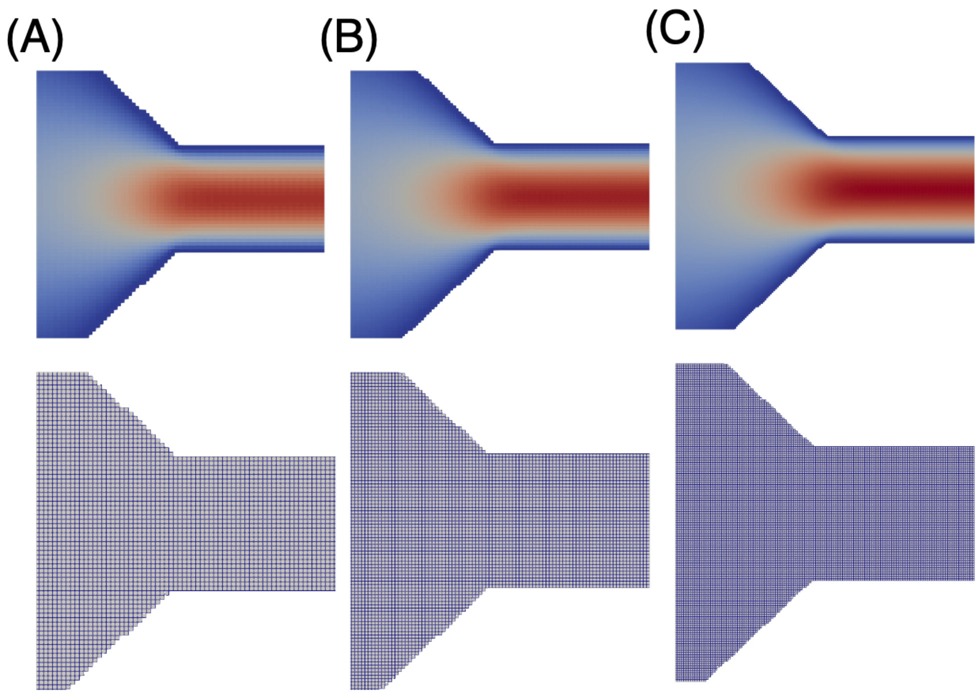

Figure 4. LBM lattice resolutions considered for the refinement studies, zoomed in on the entrance to the microfluidic constriction. The resolutions depicted in Figures A-C correspond to 0.2, 0.15, and 0.1μm lattices spacings. The upper-most image in each figure gives contours of the velocity magnitude through the device, while the lower-most image depicts the lattice spacing.

slight difference between the passage times predicted with fluid resolutions of 0.15 and 0.1um, however at 20480 elements and above the passage times converge. With regard to the simulation results presented in the main text, the studies detailed here in the SI were performed apriori in order to determine the mesh resolutions required. Based on the results here, we consider a lattice resolution of 0.15um and an FEM mesh resolution of 20480 elements for all simulations presented in the main text. The sufficiency of this to capture the relevant physics for the present work is supported by both the results presented here as well as the good agreement with experiments presented in the main text.

While the cell passage time is the primary metric used in the main text to validate the model results, here we also study the time-dependent 3D deformation characteristics of the nucleated cell to ensure that the cell shapes converge as well. We quantify cell deformation using the Taylor deformation parameter D, defined as[4,5]:

$$D = \frac{L - B}{L + B} \tag{7}$$

where L is the instantaneous major axis of the cell and B is the instantaneous minor axis. We determine D for both the outer plasma membrane and the nucleus, and to best illustrate differences in cell shapes based with increasing resolution, here we focus on fluid resolutions of 0.2 and 0.15μm. In Figure 6 D versus time considering these resolutions, and FEM mesh resolutions of 5120, 20480, and 81920 elements. Results for the outer plasma membrane are given in Figure 6A. First, considering the 0.2μm fluid resolution (left-most figure), it can be seen that up until $t' = 0.4$ the cell shapes are in agreement for each of the FEM mesh resolutions. D then subsequently plateaus, which delineates the point at which the cell has completely entered the constriction. From this point onward there is a shape difference increasing from 5120 elements to 20480 elements, but from 20480 to 81920 the shapes are virtually identical indicating convergence. The results for 0.15μm resolution shown in the right-most figure depict the same trend, namely shape convergence for 20480 elements and higher.

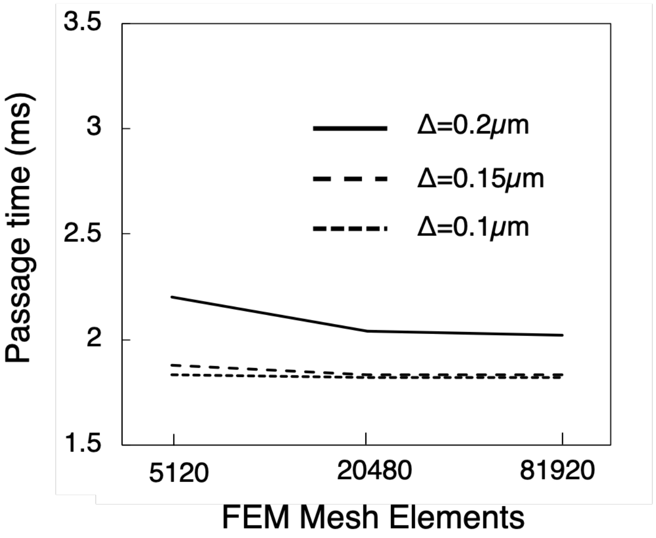

Figure 5. Passage times predicted by the nucleated cell model with different combinations of LBM fluid resolution and FEM mesh resolution. Starting with a fluid resolution of 0.2um (solid black curve), increasing the fluid resolution to 0.15um (long-dash curve) results in a different passage time regardless of the FEM mesh resolution. Increasing the fluid resolution to 0.1um (short-dash curve), the passage times can be observed to converge at an FEM mesh resolution of 20480 elements and higher.

Next we consider the time dependent shape evolution of the nucleus in Figure 6B. The results for the outer membrane shape in Figure A indicated convergence at 0.2um resolution and 20480 elements, however based on the passage time convergence trend in Figure 5 we know that this requirement is more stringent at 0.15um and 20480 elements. Agreement with this latter convergence requirement can also be observed in the cell shape characteristics by considering the nucleus. In Figure 6B the left-most image gives the evolution of D at 0.2um fluid resolution. Similar to the outer membrane trends there is a notable difference between 5120 FEM mesh elements and 20480 elements after the cell has entered the constriction. Increasing the FEM resolution from 20480 to 81920 brings the curves very close to one another, however there is a slight discrepancy which can be observed. In contrast, the results at 0.15um resolution in the right-most image show that the curves for 20480 and 81920 are virtually identical, indicating convergence for the nucleus shape. This analysis demonstrates the importance of considering the shape evolution convergence of both the outer membrane and the nucleus, and further confirms the 0.15um fluid resolution and 20480 FEM mesh elements used for the simulations described in the main manuscript text.

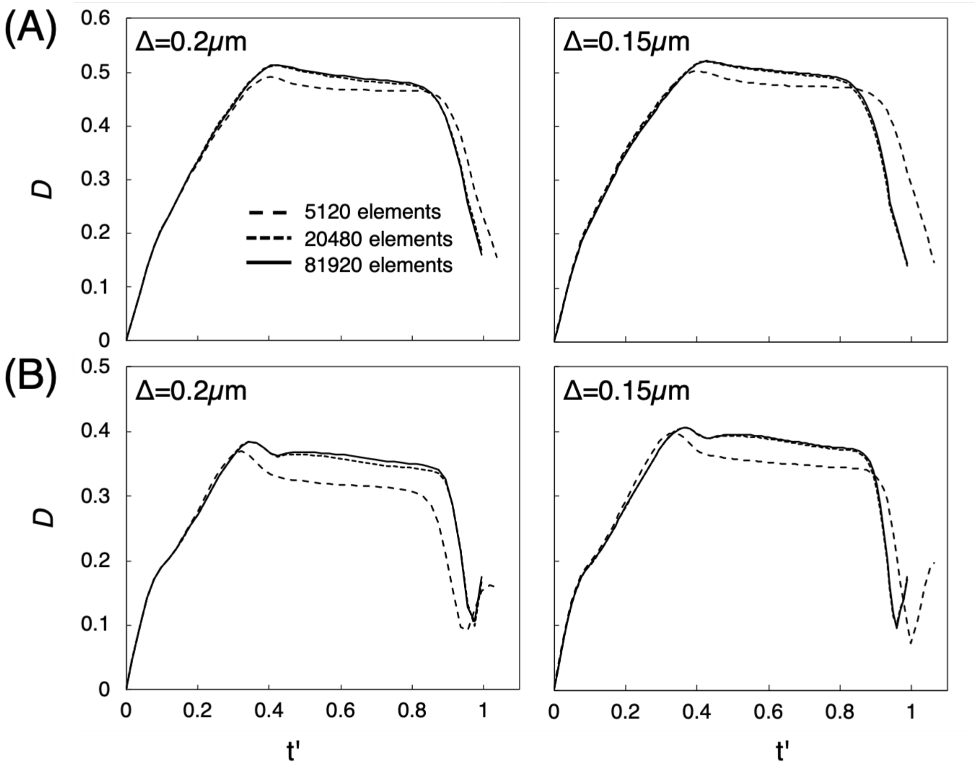

Figure 6. Cell shape evolution convergence considering both the outer plasma membrane and the nucleus. (A) the Taylor deformation parameter D for the outer membrane is plotted versus time normalized by the passage time (t'). The left-most figure is for simulations with fluid resolution of 0.2μm, while the right-most figure corresponds to 0.15μm simulations. For each, results are plotted for 5120, 20480, and 82920 elements, and convergence can be observed at 0.2μm and 20480 elements. (B) D is plotted for the nucleus versus t', for the same respective cases as given in (A). In contrast to (A), here it is observed that the time-dependent nucleus shape convergence requires a fluid resolution of 0.15um and 20480 elements. This can be see by slight difference in trends between the 20480 and 81920 element cases in the left-most figure (i.e. 0.2um resolution), while in the right-most figure (0.15um resolution) these same curves are identical. This convergence criteria based on nucleus shape is consistent with that determined based on cell passage time in Figure 5, and underscores the importance of considering the shape evolution of both membranes.

3 Main Algorithmic Components to Cell-Resolved Simulations

3.1 Lattice Boltzmann Method-Based Fluid Dynamics Solver

The foundation of the LBM fluid dynamics solver is the lattice Boltzmann equation, with this fluid-structure interaction (FSI) incorporated by means of a distributed body force[6]:

$$f_i(\mathbf{x}+\boldsymbol{c}_i\delta t, t+\delta t) = f_i(\mathbf{x},t) - \frac{1}{\tau}\left(f_i(\mathbf{x},t) - f_i^{eq}(\mathbf{x},t)\right) - h_i(\mathbf{x},t)\,\delta t \tag{8}$$

Here, f_i is the particle distribution function, f_i^{eq} is an approximation of the Maxwell-Boltzmann equilibrium distribution, h_i is the distributed body force, δt is the time step, and the Bhatnagar-Gross-Krook collision operator[?] is used. The distribution is relaxed toward equilibrium with time constant τ, which is related to the modeled fluid kinematic viscosity ν as:

$$\nu = c_s^2\left(\tau - \frac{\delta t}{2}\right), \tag{9}$$

where $c_s = \delta x / \left(\sqrt{3}\delta t\right)$ is the speed of sound, and δx is the physical spacing between lattice points.

The LBM is a mesoscopic approach to numerically solve the Navier-Stokes equations[7], where the fluid is represented by this particle distribution function that evolves following Eq. 8. We solve Eq. 8 on a uniform Eulerian lattice using a D3Q19 velocity discretization, and $\boldsymbol{c}_i$ is the velocity of the ith population in accordance with this scheme[6]:

$$c_i = \begin{bmatrix} 0 & 1 & -1 & 0 & 0 & 0 & 0 & 1 & -1 & 1 & -1 & 0 & 0 & 1 & -1 & 1 & -1 & 0 & 0 \\ 0 & 0 & 0 & 1 & -1 & 0 & 0 & 1 & -1 & 0 & 0 & 1 & -1 & -1 & 1 & 0 & 0 & 1 & -1 \\ 0 & 0 & 0 & 0 & 0 & 1 & -1 & 0 & 0 & 1 & -1 & 1 & -1 & 0 & 0 & -1 & 1 & -1 & 1 \end{bmatrix}, \quad (10)$$

with the standard D3Q19 weights, w_i:

$$w_0 = 1/3,\ w_{1-6} = 1/18,\ w_{7-18} = 1/36. \quad (11)$$

The macroscopic fluid density is the 0th order moment:

$$\rho = \sum_i f_i(\mathbf{x}, t) \quad (12)$$

and the momentum is the first order moment, from which we get an expression for the macroscopic velocity in the presence of a body force:

$$\boldsymbol{v} = \frac{1}{\rho}\sum_i \boldsymbol{c}_i f_i(\mathbf{x}, t) + \frac{\delta t}{2\rho}\boldsymbol{g} \quad (13)$$

where $\boldsymbol{g}$ is a body force in the form of force per volume. With the simulation technique employed here, this body force term is used to distribute the stress generated in the cell membranes as they deform to the fluid points on the Eulerian lattice. The relationship between this body force and the force distribution associated with the ith population used in Eq. 8 is determined following[8]:

$$h_i = \left(1 - \frac{1}{2\tau}\right) w_i \left[\frac{\boldsymbol{c}_i - \boldsymbol{v}}{c_s^2} + \frac{\boldsymbol{c}_i \cdot \boldsymbol{v}}{c_s^4}\boldsymbol{c}_i\right] \cdot \boldsymbol{g} \quad (14)$$

In terms of practical implementation of the LBM, and consideration with regard to deformable cell modeling as described below, there are many through and detailed works in the literature (e.g. Krüger et. al[6]) to which the reader is referred for implementation guidance.

3.2 Cell Dynamics using the Immersed Boundary and Finite Element Methods

Deformable cells are modeled within the LBM fluid dynamics solver by using the Immersed Boundary Method (IBM) to two-way couple the stresses generated in the membranes as they deform to the fluid which conveys them. These stresses are calculated using the Finite Element Method (FEM), solved on a Lagrangian grid which discretizes the cell membrane with triangular elements. A primary ingredient to the IBM which couples this Langrangian grid to the Eulerian grid (lattice) on which the LBM equation is solved is the Dirac delta function, whose discrete form we take as[9]:

$$\delta(\boldsymbol{x} - \boldsymbol{X}) = \frac{1}{64\delta x^3}\prod_{i=1}^{3}\left[1 + \cos\frac{\pi}{2\delta x}(x_i - X_i)\right] \quad (15)$$

where x_i are the fluid lattice coordinates, X_i are the vertex coordinates of the Lagrangian mesh on the cell, δx is the spacing between lattice points, and the stencil spans four Eulerian lattice points in each direction, centered on x_i. The means by which Eq. 15 facilitates the relevant information transfers between grids is described below.

The Lagrangian grid of triangular elements is created for each cell by starting with an icosahedron, and then successively refining it. Representative examples of different refinement levels are depicted in Figure 3. The

relationship between refinement level (N_{refine}) and the number of triangular elements (N_e) and vertices (N_v) is given by:

$$N_e = 20 \cdot 4^{N_{refine}} \tag{16}$$

$$N_v = 2 + \frac{N_e}{2} \tag{17}$$

Given this Lagrangian mesh, and the LBM solver described in the previous section, the basic procedure for evolving the 3D fluid field and the deformable cell membranes with time is described below.

Procedure:

1. Solve Eq. 8 to get the updated particle distribution function (f_i), which for the first timestep uses the given initial conditions and zero body force.

2. Determine the fluid velocity ($\boldsymbol{v}$) from Eqs. 12-13

3. Using Eq. 15, interpolate the Lagrangian membrane velocity ($\boldsymbol{V}$) at each vertex ($\boldsymbol{X}$):

$$\boldsymbol{V} = \int_{\mathcal{V}} \boldsymbol{v}\delta\left(\boldsymbol{x} - \boldsymbol{X}\right) d\boldsymbol{x} \tag{18}$$

   where the integral is over the fluid volume.

4. Using this membrane velocity, update each membrane vertex location using a second-order Adams-Bashforth scheme of the form:

$$X_i(t+\delta t) = X_i(t) + \delta t \left(\frac{3}{2} v_i(t) - \frac{1}{2} v_i(t-\delta t)\right) \tag{19}$$

5. At this point, the membrane as deformed. The resulting force in the membrane due to the deformation is determined using the finite element method (FEM), and Loop elements are used as subdivision surface for the force calculations[10–13].

   (a) The displacement field for an element ($\boldsymbol{X}_e$) is determined using box-spline shape functions (N_j^e) and the vertex coordinates for the element's 1-ring:

$$\mathbf{X_e} = \sum_{j \in E_j} N_j^e \boldsymbol{X}_j \tag{20}$$

   implemented in the same manner as in Cirak et. al[11].

   (b) Determine the deformation gradient tensor:

$$\mathbf{F} = \boldsymbol{A}_\alpha \otimes \boldsymbol{A}^{\mathbf{0}^\alpha} \tag{21}$$

   where $\boldsymbol{A}_\alpha$ and $\boldsymbol{A}^{0^\alpha}$ are tangent vectors calculated from X in local covariant and contravariant bases, respectively[14].

   (c) the right Cauchy-Green tensor ($\boldsymbol{C}$) and the strain invariants (I_1, I_2) are given by:

$$\mathbf{C} = \mathbf{F}^T \cdot \mathbf{F} \tag{22}$$

$$I_1 = tr(\mathbf{C}) \tag{23}$$

$$I_2 = det(\mathbf{C}) \tag{24}$$

(d) Given the strain energy function of Skalak et. al[15], determine its derivatives with respect to the strain invariants:

$$\frac{\partial W_s}{\partial I_1} = \frac{G_s}{2}(I_1+1) \tag{25}$$

$$\frac{\partial W_s}{\partial I_2} = \frac{G_s}{2}(CI_2-1) \tag{26}$$

(e) Determine the Cauchy tension tensor in contravariant form[16]:

$$\mathrm{T}^{mn} = \frac{2}{J_s}\frac{\partial W_\mathrm{s}}{\partial I_1}\mathrm{A}^{0mn} + 2J_s\frac{\partial W_\mathrm{s}}{\partial I_2}\mathrm{A}^{mn}. \tag{27}$$

where G_s is the membrane shear elasticity, C is a constant related to the area dilation modulus, and here we use $C = 100$.

(f) From the Helfrich bending energy, determine the membrane and bending stresses[12]:

$$\sigma^{mn} = \frac{k_\mathrm{b}}{2}\left(4\kappa^2\mathrm{A}^{mn} - 8\kappa\mathrm{B}^{mn}\right) \tag{28}$$

$$\mu^{mn} = \frac{k_\mathrm{b}}{2}\left(4\kappa\mathrm{A}^{mn}\right) \tag{29}$$

where k_b is the bending modulus, κ is the mean curvature, and A and B are the metric and curvature tensors, which can be computed as described in[12].

(g) Determine the volume penalty force density $(\mathbf{G}_v)$ based on the following energy conservation law:

$$W_v(t) = \frac{k_v}{2}\frac{(V(t)-V_0)^2}{V_0} \tag{30}$$

where k_v gives the volume preservation coefficient which we consider to be 0.1, $V(t)$ is the cell volume at time t, and V_0 is the initial cell volume.

(h) Calculate the surface force density $(\mathbf{G}_e)$ at each element by evaluating the surface gradient of the following quantities, as described in[12]:

$$\mathbf{G}_e = \nabla_s\cdot(T^{mn}+\sigma^{mn}+\mu^{mn}) + \mathbf{G}_v \tag{31}$$

6. Transfer the surface force densities to the vertices[13]:

$$\mathbf{G} = \frac{\mathbf{G}_e}{\int_S N_j^e \mathrm{d}S}. \tag{32}$$

7. Spread the forces from the vertices back to the Eulerian lattice using the same delta function in Eq. 15:

$$\boldsymbol{g} = \int_S \boldsymbol{G}\delta(\boldsymbol{x}-\boldsymbol{X})\,d\boldsymbol{X} \tag{33}$$

8. Calculate the force distribution per Eq. 14, to be used in Eq. 8.

3.3 Adaptive Spring-Based Cyto- and Nucleo-skeleton Model

The adaptive spring-based model developed in this work mimics the mechanical structure of the cyto- and nucleo-skeleton. We note that this model is simplistic in that it does not directly capture the more complex structures which comprise the actual interior of a cancer cell. However, this provides an additional measure beyond the membrane model alone to directly capture the resistance to deformation caused by internal skeletal networks themselves.

With this model, linear springs connect from the outer plasma membrane to the nucleus, as well as inside the nucleus between opposing locations. Springs are connected to each element of the Lagrangian mesh for the cell membranes, and the resulting forces are added to those determined by the FEM calcualtions in Eq. 31. Specifically, the force in each spring is determined from Hooke's law:

$$F = k \cdot dL \tag{34}$$

where the spring constant $k = EA/L_0$, and $dL = L(t) - L_0$ which gives the spring deformation relative to the initial reference length L_0. Additionally E is the Young's modulus and A is the elemental area associated with the spring.

As the cell undergoes complex deformation, the adaptive component to this model allows springs to adapt to the instantaneous cell curvatures to prevent unphysical behavior where the springs extend outside of the membrane surfaces, as discussed in the main text. The overall procedure to integrate this model into the membrane force calculations is described below, which includes implementation details for the adaptive spring component. Also provided below are Figures 2 and 9 from the main text to help illustrate the procedure.

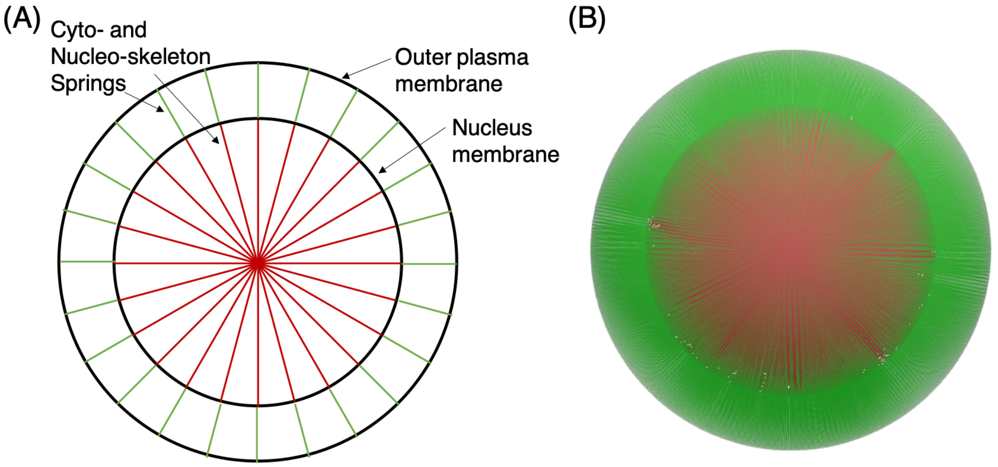

Figure 7. (Figure 2 from main text) Components of the nucleated cell with cyto- and nucleo-skeleton model. (A) Schematic depiction of each component, including the outer plasma and nucleus membranes, as well as the cyto- and nucleo-skeleton springs. Green springs connect from the outer membrane to the nucleus, and red springs connect opposing locations on the nucleus membrane. (B) 3D cell model depicting the spring configuration in the undeformed state, with 20,480 springs for each region.

Procedure:

1. Establish the spring structures at the beginning of each timestep based on the shape of the outer and nucleus membranes. Beginning with the outer membrane, determine the cytoskeleton spring structure by projecting the centroid of each triangular element inward along the element's normal vector. Determine the element intersected by this line (either on the nucleus membrane, or another element on the outer membrane), and then mark the two elements as connected by a spring. For a given element on the outer membrane, this other element to which it will connect is determined as follows:

   (a) Given an element centroid $\mathbf{x}_A$ and inward unit normal vector $\mathbf{n}_A$, loop through all other elements on the membranes and find the intersection point $\mathbf{x}_i$ on each elements plane, defined by a centroid $\mathbf{x}_B$ and unit

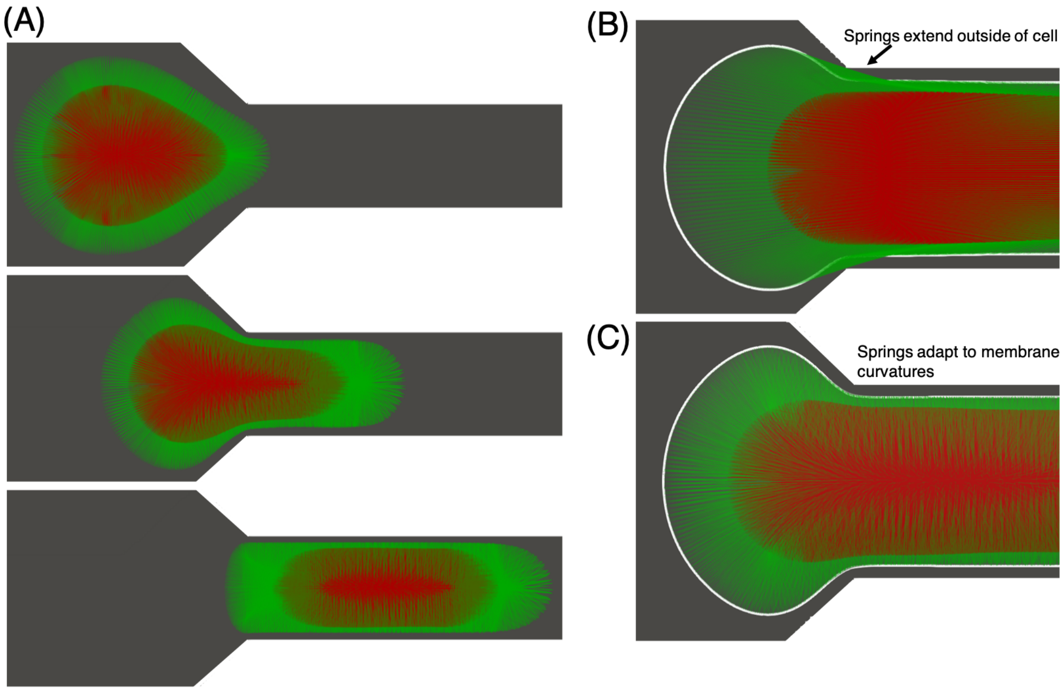

Figure 8. (Figure 9 from main text) Simulation snapshots depicting the nucleated cell model augmented by cyto- and nucleo-skeleton components deforming to enter the constricted microchannel. Cytoskeleton springs are given by green lines, and nucleoskeleton springs are given by red lines. (A) Snapshots in sequence showing the deformation associated with the process of cell entry, and the adapting structure of the springs. (B,C) Zoomed in view depicting the end of the entry process. In (B) we show how springs can extend outside of the cell due to the instantaneous membrane structures, which is remedied by implementing an adaptive technique (C) where the springs restructure based on the membrane curvatures.

normal vector $\mathbf{n}_B$:

$$d\mathbf{x} = \mathbf{x}_B - \mathbf{x}_A \tag{35}$$

$$t = \frac{\mathbf{n_B} \cdot d\mathbf{x}}{\mathbf{n_A} \cdot \mathbf{n_B}} \tag{36}$$

$$\mathbf{x}_i = \mathbf{x}_A + t\mathbf{n}_A \tag{37}$$

(b) If $t >= 0$ determine if this intersection point on the element's plane is inside the actual triangle defining the element using barycentric coordinates. Each triangle is defined by vectors $\mathbf{U}$ and $\mathbf{V}$ connecting vertices 0, 1, and 2, and vector $\mathbf{W}$ locates the intersection point:

$$\mathbf{U} = \mathbf{x}_A^1 - \mathbf{x}_A^0 \tag{38}$$

$$\mathbf{V} = \mathbf{x}_A^2 - \mathbf{x}_A^0 \tag{39}$$

$$\mathbf{W} = \mathbf{x}_i - \mathbf{x}_A^0 \tag{40}$$

The barycentric coordinates (α, β, γ) are then determined[17]:

$$\gamma = \frac{(\mathbf{U} \times \mathbf{W}) \cdot \mathbf{n}_B}{\mathbf{n}_B \cdot \mathbf{n}_B} \tag{41}$$

$$\beta = \frac{(\mathbf{W} \times \mathbf{V}) \cdot \mathbf{n}_B}{\mathbf{n}_B \cdot \mathbf{n}_B} \tag{42}$$

$$\alpha = 1 - \gamma - \beta \tag{43}$$

and by definition x_i is within the triangle if α, β, and γ are all $>= 0$ and $<= 1$. Note that this line can intersect multiple elements, but the one closest to the central element is identified as the connecting element.

2. The cytoskeleton spring structure is also comprised of springs which connect from the nucleus membrane outward to the outer membrane, and these springs are determined in a similar manner to that above, except in Step 1a the outward unit normal vector is used for $\mathbf{n}_A$.

3. Determine the nucleoskeleton spring structure by projecting the centroid of each triangular element on the nucleus membrane inward, and connect it to the element intersected by the line on the opposing side. The procedure for this is identical that above, except here the elements tested for intersection are limited to those on the nucleus membrane.

4. With the cyto- and nucleo-skeleton springs adapted to the local membrane shapes, at each timestep determine the spring length $L(t)$, and evaluate the force exerted by each spring on each element from Eq. 34.

5. Add these forces at each element to those computed per Eq. 31.

4 Simulation Data for Cell Passage Time

Output data for time dependent cell shapes which were used to determine passage time for representative cases can be downloaded **here**. This data is for L1210 cells with the nucleated cell model, and was used to determine the passage time data points in Figure 6 in the main text for the $\alpha = 15.5$ case. For each data point, simulation data is provided for the outer and nucleus membranes in .vtk format, which can be loaded in the open-source visualization software Paraview (`https://www.paraview.org/`). The passage time is determined for each simulation as the sum of the entry time and the transit time. The entry time is defined as the time from when the leading edge of the cell enters the 6μm constriction region to the time when the trailing edge of the cell enters this region. For the transit time, as discussed in the main text we model half of the constricted channel length here, and given the linear velocity of the cell once it enters the constriction region the transit time is 2 times the simulated transit time. The simulated transit time is defined as the time from when the entry time ends to the time when the trailing edge exits the constriction region.

References

1. Byun, S. *et al.* Characterizing deformability and surface friction of cancer cells. *Proc. Natl. Acad. Sci.* **110**, 7580–7585 (2013).

2. Raven, F. H. *Automatic control engineering* (McGraw-Hill, Inc., 1995).

3. Boussinesq, J. Mémoire sur l'influence des frottements dans les mouvements réguliers des fluids. *J. de mathématiques pures et appliquées* **13**, 377–424 (1868).

4. Gounley, J., Draeger, E. W. & Randles, A. Numerical simulation of a compound capsule in a constricted microchannel. *Procedia computer science* **108**, 175–184 (2017).

5. Luo, Z. Y., He, L. & Bai, B. F. Deformation of spherical compound capsules in simple shear flow. *J. Fluid Mech.* **775**, 77–104 (2015).

6. Krüger, T. *et al.* The lattice boltzmann method. *Springer Int. Publ.* **10**, 978–3 (2017).

7. Chen, S. & Doolen, G. D. Lattice boltzmann method for fluid flows. *Annu. review fluid mechanics* **30**, 329–364 (1998).

8. Guo, Z., Zheng, C. & Shi, B. Discrete lattice effects on the forcing term in the lattice boltzmann method. *Phys. Rev. E* **65**, 046308 (2002).

9. Peskin, C. S. Numerical analysis of blood flow in the heart. *J. computational physics* **25**, 220–252 (1977).

10. Loop, C. Smooth subdivision surfaces based on triangles. *Master's thesis, Univ. Utah, Dep. Math.* (1987).

11. Cirak, F., Ortiz, M. & Schröder, P. Subdivision surfaces: a new paradigm for thin-shell finite-element analysis. *Int. J. for Numer. Methods Eng.* **47**, 2039–2072 (2000).

12. Boedec, G., Leonetti, M. & Jaeger, M. Isogeometric fem-bem simulations of drop, capsule and vesicle dynamics in stokes flow. *J. Comput. Phys.* **342**, 117–138 (2017).

13. Le, D. V. Effect of bending stiffness on the deformation of liquid capsules enclosed by thin shells in shear flow. *Phys. Rev. E* **82**, 016318 (2010).

14. Green, A. E. & Adkins, J. E. Large elastic deformations (Oxford University Press, 1960).

15. Skalak, R., Tozeren, A., Zarda, R. & Chien, S. Strain energy function of red blood cell membranes. *Biophys. journal* **13**, 245 (1973).

16. Walter, J., Salsac, A.-V., Barthès-Biesel, D. & Le Tallec, P. Coupling of finite element and boundary integral methods for a capsule in a stokes flow. *Int. journal for numerical methods engineering* **83**, 829–850 (2010).

17. Heidrich, W. Computing the barycentric coordinates of a projected point. *J. Graph. Tools* **10**, 9–12 (2005).
